# Hallmarks of aging-based dual-purpose disease and age-associated targets predicted using PandaOmics AI-powered discovery engine

**DOI:** 10.18632/aging.203960

**Published:** 2022-03-29

**Authors:** Frank W. Pun, Geoffrey Ho Duen Leung, Hoi Wing Leung, Bonnie Hei Man Liu, Xi Long, Ivan V. Ozerov, Ju Wang, Feng Ren, Alexander Aliper, Evgeny Izumchenko, Alexey Moskalev, João Pedro de Magalhães, Alex Zhavoronkov

**Affiliations:** 1 Insilico Medicine Hong Kong Ltd., Hong Kong Science and Technology Park, New Territories, Hong Kong, China; 2 Department of Medicine, Section of Hematology and Oncology, University of Chicago, Chicago, IL 60637, USA; 3 School of Systems Biology, George Mason University (GMU), Fairfax, VA 22030, USA; 4 Integrative Genomics of Ageing Group, Institute of Ageing and Chronic Disease, University of Liverpool, Liverpool L7 8TX, UK; 5 Buck Institute for Research on Aging, Novato, CA 94945, USA

**Keywords:** artificial intelligence, deep learning, drug discovery, multi-omics, target identification

## Abstract

Aging biology is a promising and burgeoning research area that can yield dual-purpose pathways and protein targets that may impact multiple diseases, while retarding or possibly even reversing age-associated processes. One widely used approach to classify a multiplicity of mechanisms driving the aging process is the hallmarks of aging. In addition to the classic nine hallmarks of aging, processes such as extracellular matrix stiffness, chronic inflammation and activation of retrotransposons are also often considered, given their strong association with aging. In this study, we used a variety of target identification and prioritization techniques offered by the AI-powered PandaOmics platform, to propose a list of promising novel aging-associated targets that may be used for drug discovery. We also propose a list of more classical targets that may be used for drug repurposing within each hallmark of aging. Most of the top targets generated by this comprehensive analysis play a role in inflammation and extracellular matrix stiffness, highlighting the relevance of these processes as therapeutic targets in aging and age-related diseases. Overall, our study reveals both high confidence and novel targets associated with multiple hallmarks of aging and demonstrates application of the PandaOmics platform to target discovery across multiple disease areas.

## INTRODUCTION

Population aging is a key social, economic, and medical challenge on a global scale, which has created a major, growing need to develop interventions that target the aging process. Because age-associated changes in homeostasis are the major risk factors for the most prevalent human diseases (such as neurological, metabolic, fibrotic, and inflammatory conditions), developing interventions that target aging would also impact multiple age-related diseases and result in unprecedented health benefits [1, 2]. Importantly, the stated goal of geroscience is to extend not only lifespan but also health life expectancy, or healthspan [2], enabling wellbeing in older age, the so-called “healthy aging”. Research into longevity pharmacology has exploded in recent years with hundreds of compounds now known to extend lifespan in model organisms [3–7]. Major challenges remain, however, in translating these findings into humans, and ultimately extending lifespan and healthspan by targeting aging mechanisms.

Although the underlying molecular mechanisms of aging remain the subject of debate, several key pathways and processes have been associated with aging processes. These have been conceptualized in the hallmarks of aging composed of: Altered intercellular communications, Cellular senescence, Deregulated nutrient signaling, Epigenetic shift, Genomic instability, Impaired proteostasis, Mitochondrial dysfunction, Stem cell exhaustion, Telomere attrition [8]. The hallmarks of aging have been widely used in the field as a starting point for studies, although they are not perfect, and arguably other mechanisms such as extracellular matrix stiffness [9], retrotranspositions [10] and inflammation [11] have also been reported to play an important role in aging. These heterogeneous processes have in turn been associated with age-related diseases. For example, cellular senescence has been associated with pathologies such as cancer, type 2 diabetes, and atherosclerosis [12], as well as pulmonary, neurological, renal, hepatic, infectious, musculoskeletal, and endocrine diseases [13]. At the genetic level, there is a substantial overlap between the genetics of aging and age-associated diseases (AADs) [14]. For example, some known aging-related targets, such as mTOR, AMPK, IGFR, NF-kB, S6K, TGF-β, AT1, Fgf21, FOXO3a, SIRT1, HIF-1, NRF2, and Klotho, may also impact multiple age-associated diseases [1, 8, 15]. Therefore, given that aging is associated with mechanisms that ultimately lead to age-related comorbidities, drugs that act on targets implicated in aging may potentially reduce the severity of gerolavic (from Greek, géros “old man” and epilavís, “harmful”) diseases and preventing multimorbidity [16].

A substantial percentage of the human clinical trials, including those evaluating investigational anti-aging drugs, fail in Phase II, a phase where efficacy of the drug is tested [17, 18]. This poor success is in part due to inadequate target choice and the inability to identify a group of patients who will most likely respond to specific agents. This challenge is further complicated by the differences in biological age of the patients, as importance of therapeutic targets varies between the age groups. Hence, identifying potential targets that are implicated in multiple age-associated diseases, and also play a role in the basic biology of aging, may have substantial benefits.

Given the large number of datasets being generated, data-driven approaches (such as artificial intelligence [AI] and machine learning) are becoming instrumental across various fields in biology, including biomarker discovery and target prediction in aging [19]. Indeed, a number of studies by our group and others have employed computational and machine learning analysis to identify new candidates in the context of aging and AADs. These approaches have led to the detection of disease-related genes, caloric restriction genes, and longevity drugs [20–24]. The application of AI in the pharmaceutical industry also aims to reduce the tremendous amount of cost and time conventionally needed to discover new therapeutic targets in various diseases. There are multiple philosophies for the formulation of disease hypothesis, prioritization of pathways implicated in a disease, and selection of promising therapeutic targets. Multiple data types can be used for target discovery including text, imaging, and omics. In recent years, machine learning, and especially deep learning technologies, are rapidly increasing in popularity for target discovery. Advanced signaling pathway modeling such as iPANDA [25] and deep neural networks were used to identify promising protein targets driving complex biological processes implicated in cancer and other diseases [26], drug repurposing [27], and geroprotector discovery [28, 29]. Many of these approaches were implemented in PandaOmics, an industrial target discovery engine. Recently, PandaOmics, has successfully identified and nominated novel targets for idiopathic pulmonary fibrosis (IPF) and kidney fibrosis [30–32].

**Figure 1:**
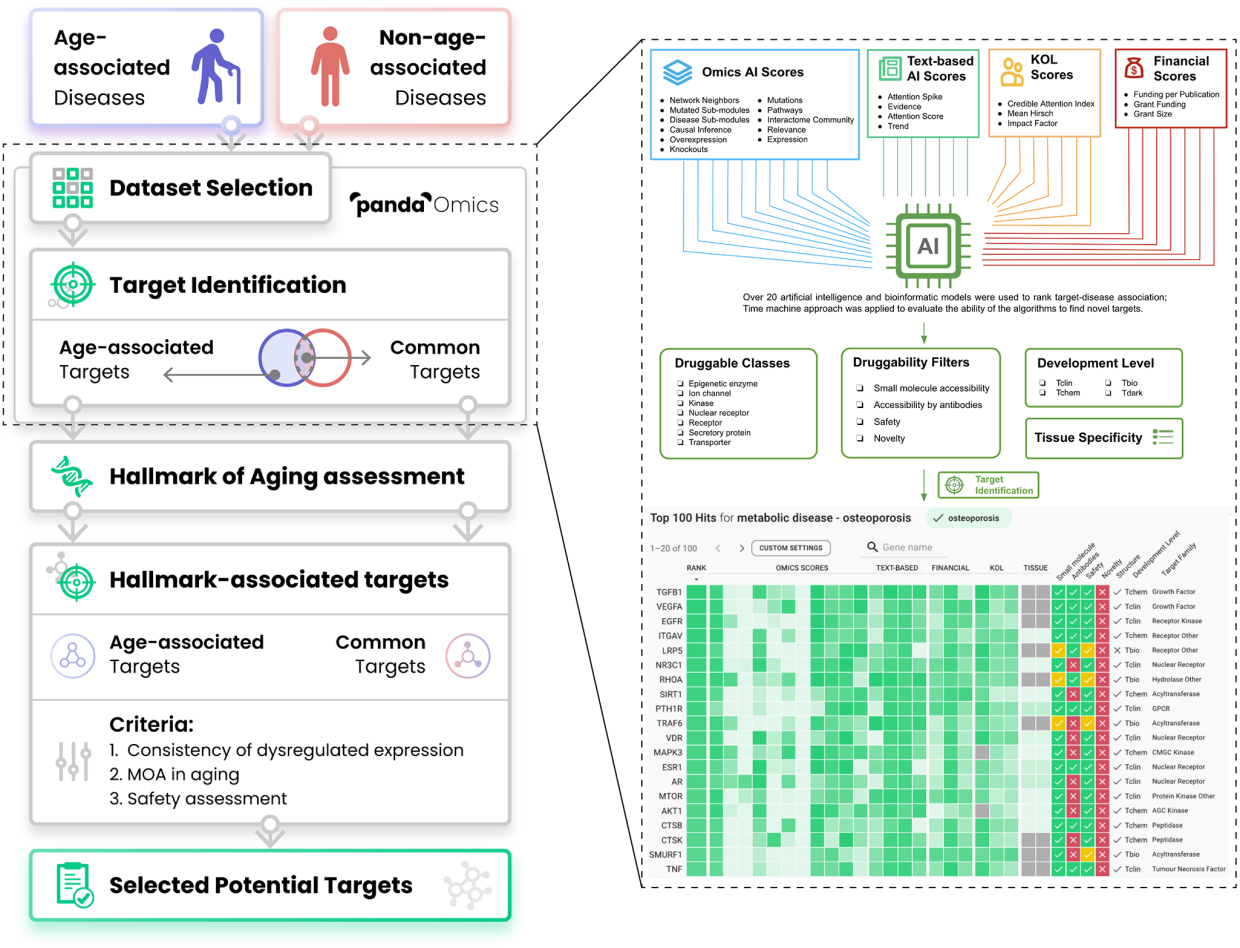
**Workflow of the present study.** Thirty-three diseases were separated into either age-associated diseases (AADs) or non-age-associated diseases (NAADs) based on the impact of age on the risk of the disease’s onset. Their corresponding transcriptomic datasets were retrieved from public repositories and processed by PandaOmics. Age bias between case and control groups has been considered during dataset selection. With multiple levels of novelty settings, targets implicated in AADs and NAADs were identified by ‘PandaOmics - target identification’. PandaOmics prioritized targets for one disease and refined the targets based on several flexible druggability filters. The target-disease associations were ranked according to over 20 artificial intelligence and bioinformatics models ranging from Omics AI scores, Text-based AI scores, Finance scores to KOL scores. Target identification was performed independently for each disease. Top-ranked targets shared by both disease categories were regarded as common targets, while targets unique to AADs were defined as age-associated targets (AAD targets). All common targets and AAD targets were subjected to the hallmarks of aging assessment by searching the literature for their evidence in modulating longevity or longevity pathways. To propose potential targets with a dual role in anti-aging and disease treatment, hallmark-associated targets were further evaluated based on their expression profiles across AADs, mechanism of action, and safety. A total of 9 targets were selected, with three levels of novelty. Abbreviation: KOL: Key opinion leader.

This platform utilizes advanced deep learning models and AI approaches to predict the target genes associated with a given disease through a combination of Omics AI scores, Text-based AI scores, Finance scores, and Key opinion leader (KOL) scores (Figure 1), and is currently being employed in both academic and industry settings. The algorithm also allows the prioritization of protein targets for novelty, confidence, commercial tractability, druggability, safety, and other key properties that drive target selection decisions. The integrated omics database consists of a vast amount of published systems biology data, spanning over 1,500 diseases and 10,000 disease subtypes. The database includes approximately 1.9 trillion data points derived from over 10 million samples with microarrays, RNA sequencing, proteomes, and methylomes, among other data types. PandaOmics’ text database embeds information from over 40 million documents, including patents, grants (that amount to over $2 trillion in funding), publications, clinical trial results, and company reports, among other text-based sources.

In this study, we used PandaOmics to identify a list of potential aging-associated therapeutic targets across various AADs that may be used for drug discovery. We successfully established and validated our unique approach with the application of varied target identification and prioritization techniques offered by PandaOmics and downstream analyses, yielding a list of dual-purpose targets associated with both the aging process and AADs. First, a list of 145 aging-related targets was generated upon the hallmarks of aging assessment. Subsequently, we further narrowed down the number of potential candidates to a total of 9 highly promising therapeutic targets associated with the aging process and AADs based on multiple selection criteria.

## RESULTS

### Discovery of targets implicated in multiple age-associated diseases

**Figure 2:**
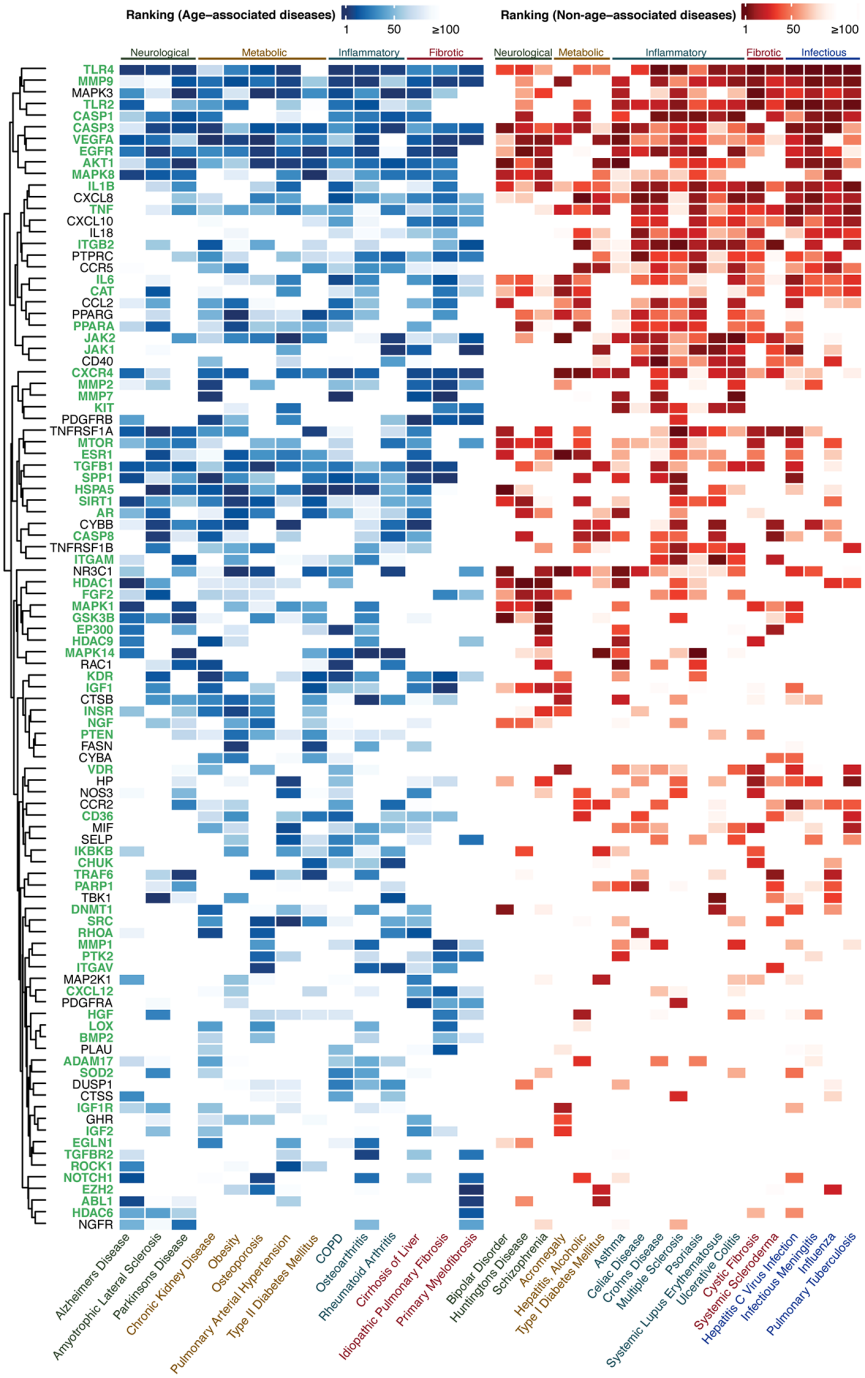
**Ranking of the top-100 gene set for AADs under high confidence settings.** The ranking of the targets in AADs and NAADs are colored in blue-white and red-white thermal scales respectively. High color intensity stands for high ranking. The lowest ranking was capped at 100. Targets associated with the hallmark(s) of aging are labeled in green. Abbreviation: COPD: Chronic obstructive pulmonary disease.

A combined list of 484 high confidence, 448 medium novel, and 381 highly novel targets were generated from the lists of top-100 targets prioritized by PandaOmics in each of the AADs (Supplementary Figure 1). The top-100 targets for three levels of novelty settings were selected by (1) the occurrence of the target genes in 14 AADs (Supplementary Figure 2) and (2) the average ranking of the target gene in its corresponding disease (Supplementary Table 1). The same approach was applied to non-age-associated diseases (NAADs). Diseases selected for this study and their corresponding disease classes were listed in Table 1. Only the top-100 genes of this combined list from AADs were subjected to the hallmarks of aging assessment by finding their corresponding evidence in modulating longevity or aging pathways in literature and expression analysis. Under high confidence settings, the top genes identified were *CASP3*, *VEGFA*, and *MMP9*, which were highly ranked in all of the 14 AADs (Figure 2 and Supplementary Figure 2A). *LYN* was the top gene identified under medium novelty settings, which was also highly implicated in all AADs (Supplementary Figures 2B and 3). For high novelty settings, *PPP2CB*, *CDC34*, *FES*, *RHOF*, and *RAB24* were the top-ranked genes in 12 out of 14 AADs (Supplementary Figures 2C and 4). The top-ranked genes shared by both AADs and NAADs were regarded as common targets, while those genes unique to AADs were defined as age-associated targets, or AAD targets (Venn diagram, Figure 1). Intersecting the two lists of genes obtained from AADs and NAADs resulted in 42 AAD targets in high confidence setting (Supplementary Figure 5A). The remaining 58 genes were considered as common targets. For medium and high novelty settings, 37 and 29 AAD targets were identified, respectively (Supplementary Figure 5B–5C).

**Table 1:** List of diseases and datasets employed.

**Disease**	**Disease class**	**Number of comparisons**
***Age-associated diseases (14 diseases, 87 comparisons)***		
Alzheimer's disease	Neurological	12
Amyotrophic lateral sclerosis	Neurological	10
Chronic kidney disease	Metabolic	7
Chronic obstructive pulmonary disease	Inflammatory	6
Cirrhosis of liver	Fibrotic	5
Idiopathic Pulmonary Fibrosis	Fibrotic	11
Obesity	Metabolic	10
Osteoarthritis	Inflammatory	5
Osteoporosis	Metabolic	2
Parkinson's disease	Neurological	4
Primary myelofibrosis	Fibrotic	2
Pulmonary arterial hypertension	Metabolic	5
Rheumatoid Arthritis	Inflammatory	4
Type II diabetes mellitus	Metabolic	4
***Non-age-associated diseases (19 diseases, 126 comparisons)***		
Acromegaly	Metabolic	2
Asthma	Inflammatory	13
Bipolar disorder	Neurological	4
Celiac disease	Inflammatory	3
Crohn's disease	Inflammatory	8
Cystic fibrosis	Fibrotic	5
Hepatitis, alcoholic	Metabolic	3
Hepatitis C virus infection	Infectious	2
Huntington's disease	Neurological	5
Infectious meningitis	Infectious	3
Influenza	Infectious	5
Multiple sclerosis	Inflammatory	11
Psoriasis	Inflammatory	11
Pulmonary tuberculosis	Infectious	7
Schizophrenia	Neurological	4
Systemic lupus erythematosus	Inflammatory	9
Systemic scleroderma	Fibrotic	6
Type I diabetes mellitus	Metabolic	12
Ulcerative colitis	Inflammatory	13

### Genes implicated in AADs are associated with the hallmarks of aging

**Figure 3:**
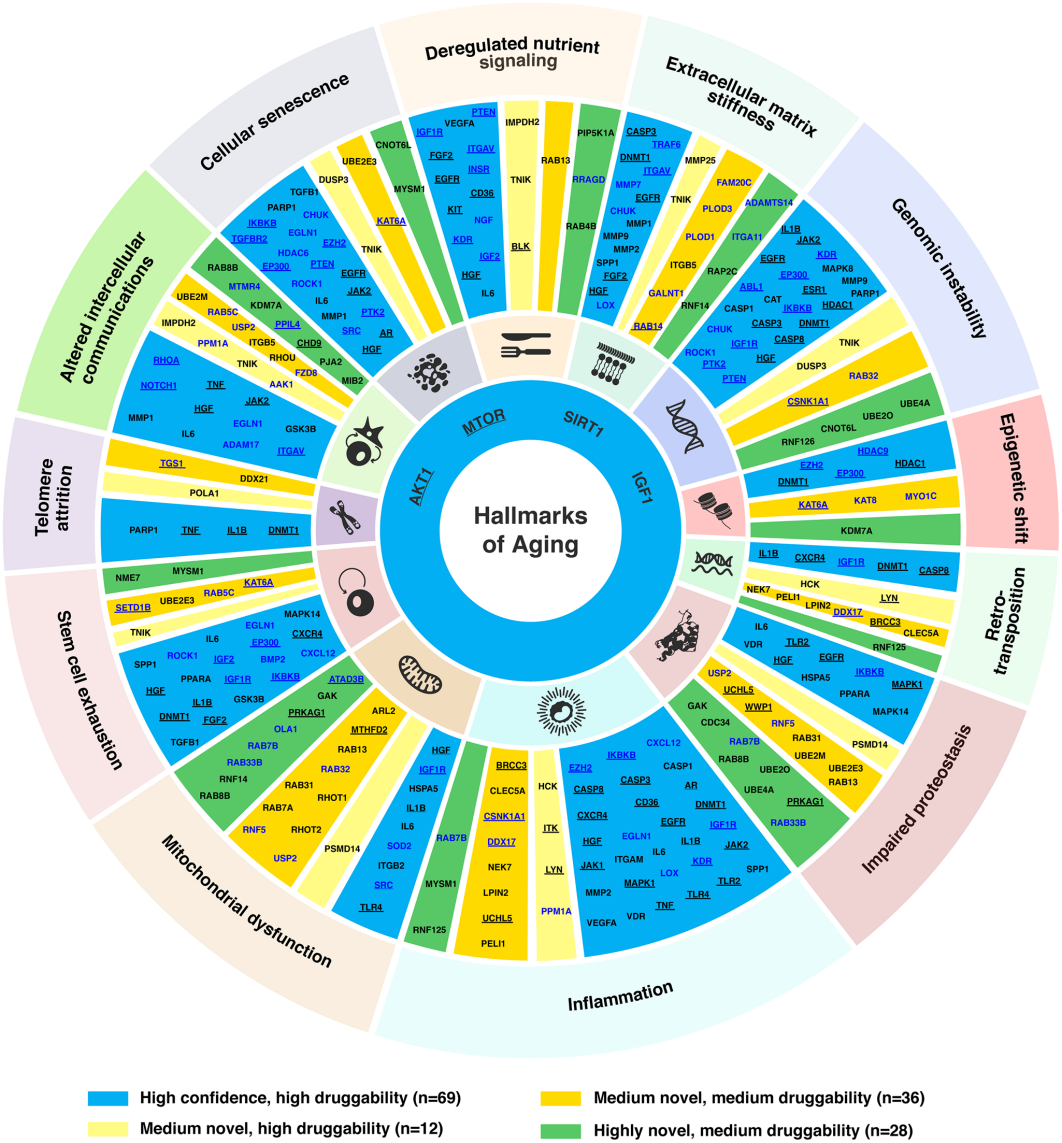
**Targets associated with hallmarks of aging.** Age-associated targets and common targets (*n* = 145) were mapped to the corresponding hallmark(s) of aging based on the literature. For novel targets, their participating pathways were also used for the assessment of their association with the hallmark(s) of aging. The four targets connected to all hallmarks (*AKT1*, *MTOR*, *SIRT1* and *IGF1*) are shown in the inner circle of the plot. The target names are labeled in blue for age-associated targets, and black for common targets. Targets annotated as cancer driver genes in the NCG7.0 database are underlined.

In our analysis, 300 genes identified under three different novelty settings were subjected to a literature review (see Materials and Methods) for their association with the hallmarks of aging. Their corresponding roles in aging processes were summarized in Supplementary Table 2. In total, 145 genes (69 high confidence, 48 medium, and 28 highly novel targets) were linked to at least one aging hallmark (Figure 3, Supplementary Table 2). The most frequently associated aging hallmark was inflammation (*n* = 48), followed by genomic instability (*n* = 35), altered intercellular communications (*n* = 33), mitochondrial dysfunction (*n* = 32), impaired proteostasis (*n* = 31) and extracellular matrix stiffness (*n* = 30). Eighty-six genes (including several well-known aging-associated genes) were found to be associated with more than one hallmark. In particular, *MTOR*, *SIRT1*, *IGF1*, and *AKT1* were associated with all hallmarks of aging, due to their wide range of interactions with aging-associated pathways. In addition, *IGF1R* was linked to deregulated nutrient signaling, genomic instability, inflammation, mitochondrial dysfunction, retrotranspositions, and stem cell exhaustion. Whereas *HGF* was associated with all hallmarks except epigenetic shift, retrotranspositions, and telomere attrition. Moreover, some novel targets were also identified to be associated with multiple hallmarks of aging. For example, *MYSM1* was associated with cellular senescence, inflammation, and stem cell exhaustion; *KAT6A* was associated with cellular senescence, epigenetic shift, and stem cell exhaustion; *UBE2E3* was linked to cellular senescence, impaired proteostasis, and stem cell exhaustion; *RAB7B* was linked to impaired proteostasis, inflammation, and mitochondrial dysfunction; whereas *RAB8B* and *USP2* were related to altered intercellular communications, impaired proteostasis, and mitochondrial dysfunction. Furthermore, a recently proposed hallmark of aging, extracellular matrix stiffness, was associated with 30 target genes identified by PandaOmics in this study, consisting of 18 high confidence (*AKT1*, *CHUK*, *CASP3*, *DNMT1*, *EGFR*, *FGF2*, *HGF*, *IGF1*, *ITGAV*, *LOX*, *MMP1*, *MMP2*, *MMP7*, *MMP9*, *MTOR*, *SIRT1*, *SPP1*, *TRAF6*), 8 medium novel (*FAM20C*, *GALNT1*, *ITGB5*, *MMP25*, *PLOD1*, *PLOD3*, *RAB14*, *TNIK*), and 4 highly novel targets (*ADAMTS14*, *ITGA11*, *RAP2C*, *RNF14*). Among the 145 genes associated with hallmarks of aging, 55 genes are known cancer drivers (Figure 3) [33], pointing to the aging components underlying cancer pathogenesis.

### Genes consistently dysregulated in multiple AADs were implicated as potential dual-purpose targets

**Figure 4:**
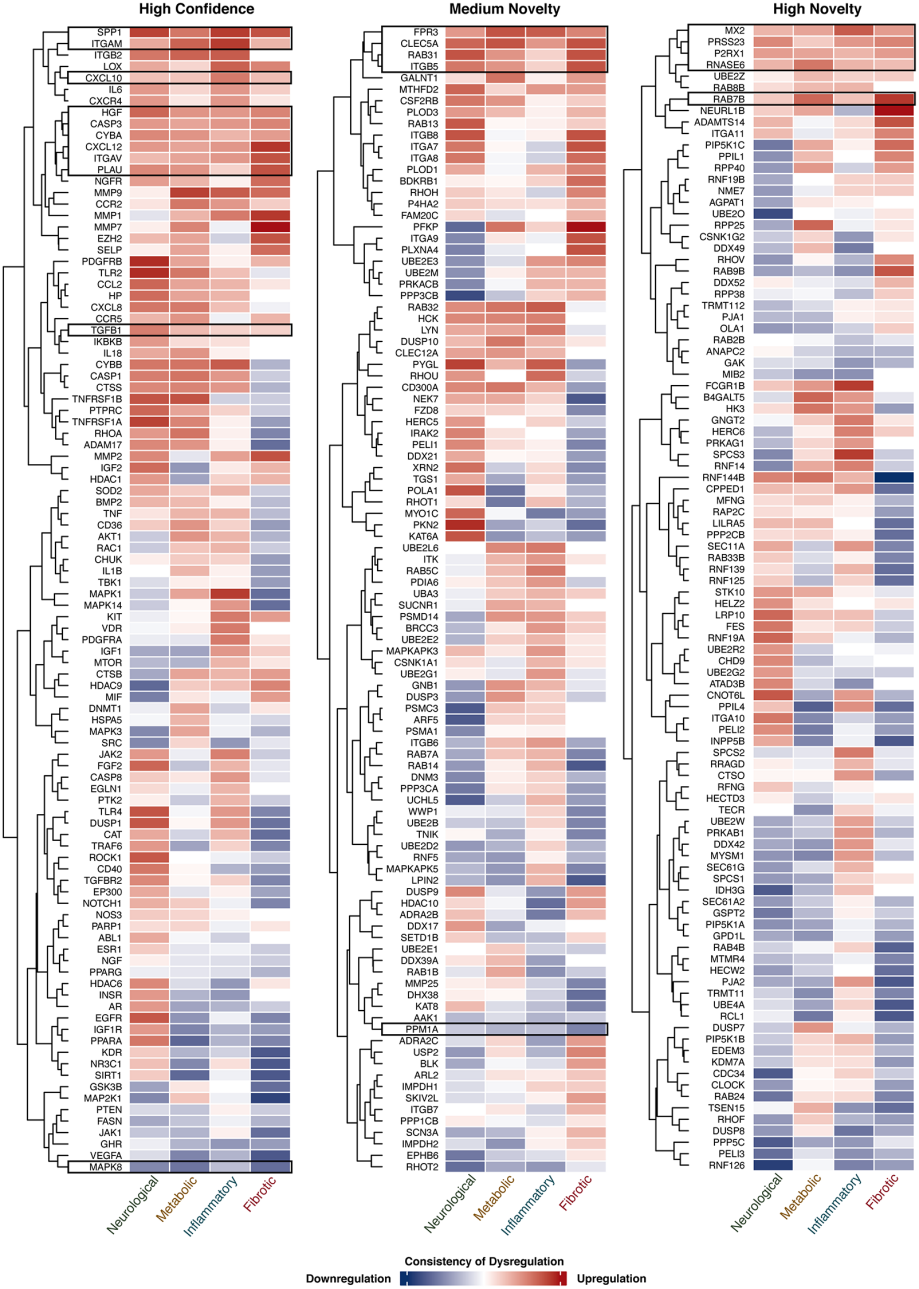
**Expression of target genes in 4 AAD classes.** The consistency of gene dysregulation in each disease class is indicated by the thermal scale, with red standing for upregulation and blue for downregulation. The color intensity indicates the level of consistency. Target genes consistently dysregulated (≥60% of comparisons) in 4 AAD classes in a unidirectional manner are shown in the black boxes.

To study the dysregulation state of genes identified under three different novelty settings, their consistency of expression change in each AAD class was summarized in Figure 4. Genes that were consistently dysregulated in two or more disease classes in a unidirectional manner were selected for further analysis. For high confidence targets, 52 genes were selected, of which 10 (*CASP3*, *CXCL10*, *CXCL12*, *CYBA*, *HGF*, *ITGAM*, *ITGAV*, *PLAU*, *SPP1,* and *TGFB1*) were consistently upregulated while *MAPK8* was the only gene that was downregulated in all disease classes; 24 genes were upregulated and 8 were downregulated in 3 disease classes. Forty-four medium novel targets were selected, with 4 genes (*CLEC5A*, *FPR3*, *ITGB5,* and *RAB31*) and *PPM1A* being upregulated and downregulated in all disease classes, respectively; 15 genes were upregulated and 10 were downregulated in 3 disease classes. For highly novel targets, 5 of the 45 genes (*MX2*, *P2RX1*, *PRSS23*, *RAB7B,* and *RNASE6*) were upregulated in all classes; 6 genes were upregulated while 21 were downregulated in 3 disease classes. As described above, upon the hallmarks of aging assessment, 145 genes were considered as potential aging-related targets. Here, these genes were further selected with reference to their expression patterns, and a subset of the candidates was considered as potential dual-purpose targets for subsequent analysis (Supplementary Figure 1).

### Validation by intersecting the AI-derived targets with well-known aging-associated genes

The significance of the mTOR, the insulin/IGF, and the sirtuin pathways in longevity has been extensively reported, delineating their critical roles in counteracting multiple hallmarks of aging to delay the aging process or to extend lifespan [8, 34, 35]. Remarkedly, Food and Drug Administration (FDA)-approved mTOR inhibitor, rapamycin, was demonstrated to slow down aging and AADs in both preclinical settings and clinical trials [36, 37]. mTOR regulates several hallmarks of aging including nutrient sensing, stem cell exhaustion, proteostasis, and cellular senescence [38]. Upon insulin/IGF receptor activation following the insulin/IGF1 binding, mTOR, a nutrient sensor, regulates cellular functions linked to proliferation, growth, and survival via Akt-mediated activation. Increased insulin sensitivity favored lifespan extension. For example, growth hormone receptor (GHR)-knockout mice showed higher sensitivity to insulin, decelerated senescence, and displayed more phenotypic features related to anti-aging [39]. In addition, activation of SIRT1 suppressed aging by ensuring telomere integrity [40, 41], antagonizing oxidative stress [42, 43], regulating nutrient signaling [8] and maintaining proteostasis [44, 45].

Our approach utilizing PandaOmics identified a set of well-known aging-associated genes that are part of the mTOR, insulin/IGF and sirtuin family signaling (including *IGF1*, *IGF1R*, *AKT1*, *MTOR,* and *SIRT1*), strongly supporting the validity of this promising method for the identification of aging-associated genes. The aging-associated genes listed above were identified as common targets, suggesting their relevance in both aging and other diseases as well as their involvement in multiple signaling networks. It is worth noting that aging genes such as *FOXO* that did not meet the criteria for druggability (see Materials and Methods) were filtered out. To further evaluate whether our approach could identify aging-related targets with potential clinical relevance, the 100 high confidence targets were compared with a pool of well-known aging-associated genes curated from http://ClinicalTrials.gov (focusing on the treatment of aging), publication, http://Geroprotectors.org [46] (Supplementary Tables 3–5) and aging gene database, GenAge [47]. Fourteen high confidence targets (*ABL1, AR, ESR1, GHR, IGF1, IGF1R, KIT, MAPK14, MTOR, NR3C1, PDGFRB, SIRT1, SRC* and *VDR*) were identified in the pool of 62 genes procured from the aging trials (expected [Exp] = 1.10, fold = 12.70, *p* = 1.79E-12, Supplementary Table 3). Twenty- four high confidence targets (*AKT1, CASP3, CAT, CHUK, DNMT1, EGFR, HDAC9, IGF1, IGF1R, IL1B, IL6, JAK2, MAPK8, MMP1, MMP2, MMP9, MTOR, PPARA, PTEN, SIRT1, SOD2, TGFB1, TGFBR2* and *TNF*) were identified in the pool of 48 genes procured from publications (Exp = 0.85, fold = 28.13, *p* = 1.21E-30, Supplementary Table 4). While seven high confidence targets (*CASP1, CASP3, CHUK, ESR1, HSPA5, IKBKB* and *MTOR*) were further identified in the pool of 52 genes procured from geroprotectors (Exp = 0.92, fold = 7.57, *p* = 3.15E-5, Supplementary Table 5). This significant enrichment might indicate the potential clinical relevance of our AI-derived targets in treating aging-related processes and AADs. Moreover, significant overrepresentation was also observed in 38 high confidence targets that were overlapped with 149 aging-associated genes obtained from the benchmark aging gene database, GenAge (Exp = 2.65, fold = 14.35, *p* = 1.28E-35), further validating the approach used in this study.

### Linking the AI-derived targets to aging-associated pathways by pathway enrichment analysis

**Figure 5:**
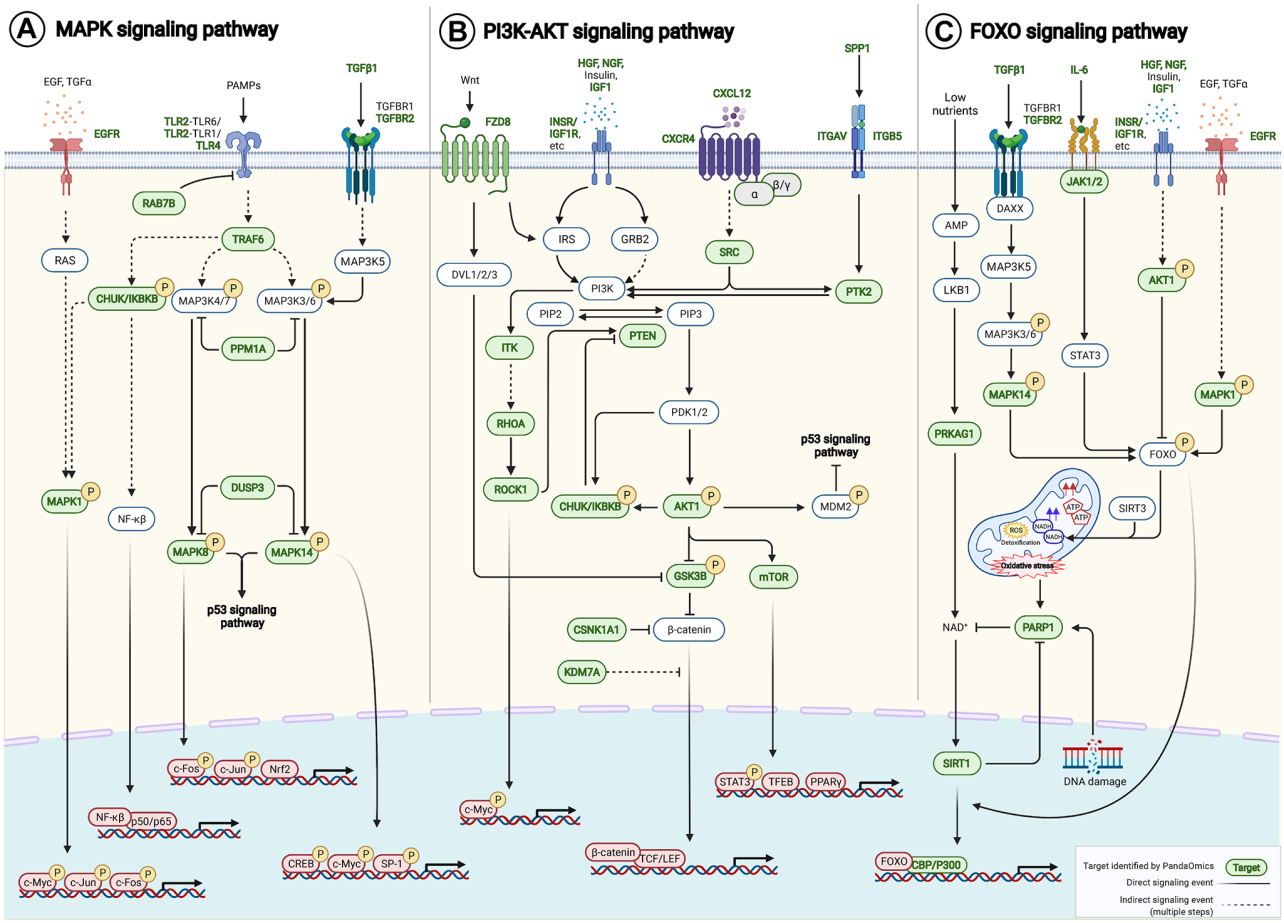
**AI-derived targets crosstalk to aging-associated signaling pathways.** Pathway enrichment analysis was performed on our 145 AI-derived targets based on KEGG PATHWAY Database. (**A**) MAPK signaling pathway (hsa04010), (**B**) PI3K-AKT signaling pathway (hsa04151) and (**C**) FOXO signaling pathway (hsa04068) were among the top 10 enriched pathways that were known to be associated with aging. Forty-six AI-derived targets were involved. Target-target interactions were identified in the contexts of pathways and networks retrieved from KEGG PATHWAY Database and literature (Supplementary Table 8). Abbreviation: PAMPs: Pathogen-associated molecular patterns.

Pathway enrichment analysis was performed on 145 AI-derived targets using Kyoto Encyclopedia of Genes and Genomes (KEGG) PATHWAY Database [48]. Genes were mapped to 225 KEGG pathways, of which 151 were significantly enriched (*p* < 0.05) (Supplementary Table 6) with 110 of the 145 potential targets. PI3K-AKT signaling pathway (hsa04151), MAPK signaling pathway (hsa04010) and FOXO signaling pathway (hsa04068) were in the top 10 enriched signaling axes known to be associated with aging. Notably, the AI-derived targets crosstalk with multiple key aging-associated pathways, such as those regulated by MAPK, PI3K-AKT and FOXO signaling networks (Figure 5), consequently contributing to modulating apoptosis, autophagy, cell proliferation, cell survival, DNA repair, epigenetic alteration, extracellular matrix organization, inflammation, mitochondrial maintenance, stemness and telomere maintenance (Supplementary Table 2).

### AI-derived targets demonstrate dual roles in aging and AADs

The dual-purpose candidates were selected under the considerations of hallmarks of aging assessment (Supplementary Table 2), expression analysis (Figure 4), ranking calculated by PandaOmics, safety assessment, clinical trial status and druggability, yielding a list of 9 potential candidates (Table 2). Selected promising high confidence targets and novel targets are discussed below.

**Table 2:** List of prioritized targets.

**Target** ^1^	**Protein family**	**Hallmarks of aging**	**Dysregulation in AAD classes**	**Role in aging**	**Drugs in clinical trials**	**Severe toxicity** ^3^	**Reference**
***High confidence***							
***CXCL12***	Cytokine	Inflammation, Stem cell exhaustion	**ALL** Upregulated	CXCL12 is an aging-upregulated gene and a mediator of the crosstalk between vascular cells and many brain cell types (pro-aging; therapy approach: antagonist)	Tinzaparin (phase 4)	No evidence	[94]
***SPP1***	Chemokine	Extracellular matrix stiffness, Inflammation, Stem cell exhaustion	**ALL** Upregulated	Age-dependent increase in SPP1 levels inhibited skeletal muscle regeneration (pro-aging; therapy approach: antagonist)	ASK-8007 (phase ½)	No evidence	[95, 96] NCT00411424
***Medium novel***							
***ITGB5***	Receptor	Altered intercellular communications, Extracellular matrix stiffness	**ALL** Upregulated	ITGB5 is a TGF-β activator. TGF-β signaling, being downstream of other signals, was shown to repress body size as well as lifespan *in vivo* (pro-aging; therapy approach: antagonist)	Cilengitide (phase 3)	No evidence	[97] NCT00689221
*PPM1A*	Esterase	Deregulated nutrient signaling, Inflammation	**ALL** Downregulated	PPM1A stimulated macrophages to produce TNF through TLR4 (anti-aging; therapy approach: agonist)	No	No evidence; absence in DEG	[98]
***Highly novel***							
*RAB7B*	Hydrolase	Impaired proteostasis, Inflammation, Mitochondrial dysfunction	**ALL** Upregulated	RAB7B negatively regulated TLR4 signaling in macrophages and autophagic flux as well as prevented inflammation and autophagy upon damage (anti-aging^2^; therapy approach: agonist)	No	No evidence; absence in DEG	[99]
***ADAMTS14***	Peptidase	Extracellular matrix stiffness	Upregulated in neurological and fibrotic diseases	ADAMTS14 is responsible for the degradation of ECM collagen. During aging, fibroblast-ECM interactions become disrupted due to the fragmentation of collagen fibrils. Fibroblasts synthesized fewer ECM proteins and more matrix-degrading metalloproteinases (pro-aging; therapy approach: antagonist)	No	No evidence, absence in DEG	[100]
*KDM7A*	Oxidoreductase	Altered intercellular communications, Genome instability	Downregulated in neurological and fibrotic diseases	Age-related neural dedifferentiation might contribute to many cognitive abilities decline with age. KDM7A regulated neural differentiation through FGF4, and was associated with Wnt signaling (anti-aging; therapy approach: agonist)	No	No evidence	[101, 102]
*MYSM1*	Peptidase	Cellular senescence, Inflammation, Stem cell exhaustion	Downregulated in neurological, fibrotic and metabolic diseases	MYSM1 functionally reduced cellular senescence and the aging process. MYSM1 deficiency promoted the aging process and decreased lifespan while its overexpression inhibited the aging process and increased lifespan *in vivo*. (anti-aging; therapy approach: agonist)	No	No evidence	[103]
*MTMR4*	Esterase	Altered intercellular communications	Downregulated in neurological, fibrotic and metabolic diseases	Skeletal muscle atrophy accompanies many chronic disease states and normal aging (anti-aging; therapy approach: agonist)	No	No evidence	[104]

Note: ^1^Targets selected for comprehensive target review are in **BOLD**. ^2^Based on its mechanism of action i.e., protective role. ^3^Database of Essential Gene (DEG) is freely accessible from the website http://tubic.tju.edu.cn/deg.

### CXCL12

**Figure 6:**
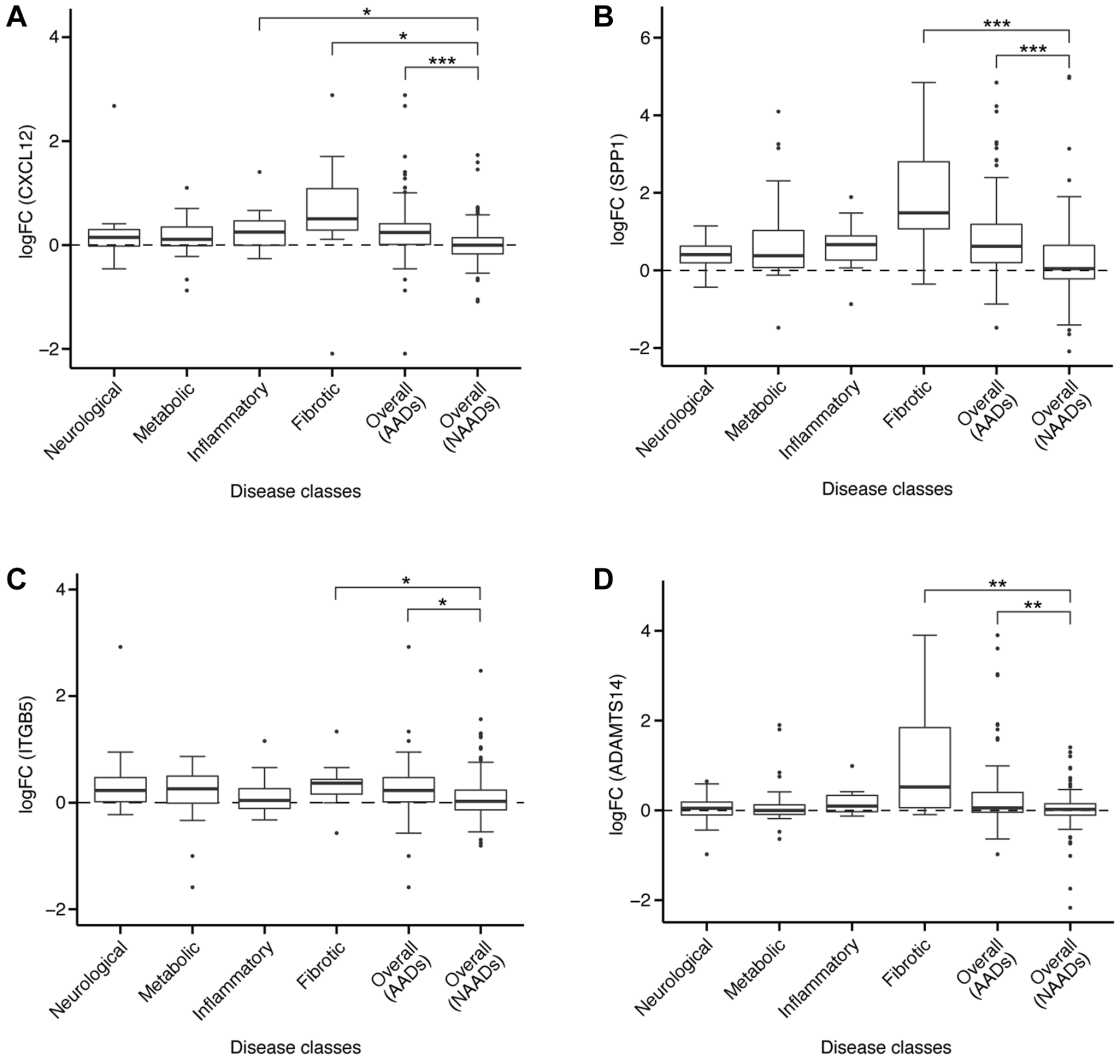
**Expression of target genes in different diseases.** The logFC of gene expression were shown for (**A**) *CXCL12*, (**B**) *SPP1*, (**C**) *ITGB5*, or (**D**) *ADAMTS14* in AADs and NAADs. For each gene, comparisons of the logFC value were conducted between NAAD and each of the AAD classes, with significant difference indicated by asterisks (two-tailed *t*-test, ^*^p < 0.05, ^**^p < 0.01, ^***^p < 0.001).

Aging was associated with elevated levels of proinflammatory cytokines, consequently leading to a decrease in mesenchymal stem cell (MSC) ability to regenerate and differentiate in inflammatory conditions [49]. Despite rescuing oxidative stress-induced hematopoietic stem cell (HSC) damage at the mitochondrial level, C-X-C motif chemokine ligand 12 (CXCL12) acted as a proinflammatory cytokine [50]. *CXCL12* was uniformly upregulated in more than 70% comparisons in all four AAD classes i.e., neurological, metabolic, inflammatory, and fibrotic diseases (Figure 6A). In general, the log-fold change (logFC) of *CXCL12* in AADs was significantly higher than in NAADs (*p* < 0.001, Figure 6A). Accumulating evidence demonstrates that CXCL12 upregulation was implicated in AADs including IPF [51], rheumatoid arthritis (RA) [52], and amyotrophic lateral sclerosis (ALS) [53]. Consistent with our findings, upregulation of CXCL12 was suggested to promote migration and proliferation of human lung fibroblast in IPF as well as to enhance monocytes infiltration into the synovial tissue in RA [51, 52]. Treatment with an antagonist of CXCR4, the receptor for CXCL12, extended lifespan, improved motor function, and led to weight loss in ALS *in vivo* [54]. Aging-associated degenerative diseases such as osteoporosis were linked to dysfunctional stem cell differentiation and a decline in the regenerative capacity of musculoskeletal stem cells, resulting from the secretion of pro-inflammatory cytokines such as CXCL12 [49]. Tinzaparin, a CXCL12 inhibitor, is an FDA-approved drug for the treatment of deep vein thrombosis and pulmonary embolism, which are considered as AADs. Taken together, suppression of CXCL12 is one of the potential therapeutic approaches that may be considered towards slowing down aging-associated processes and preventing the onset of AADs.

### SPP1

Secreted phosphoprotein 1 (SPP1) functions as Th1 cytokine and is a secreted matrix glycoprotein located in bone and produced by osteoblasts, osteocytes, other hematopoietic cells, or immune cells [55]. *SPP1* was uniformly upregulated in more than 80% comparisons in all four AAD classes, with significantly higher logFC in AADs than in NAADs (*p* < 0.001, Figure 6B). It was suggested that SPP1 may aggravate neurodegenerative, auto-immune, and inflammatory conditions. For example, SPP1 expressed by fast fatigue-resistant or slow motor neurons contributed to the second-wave neurodegeneration in ALS *in vivo* [56]. In addition, elevated levels of SPP1 in cerebrospinal fluid of subjects with Parkinson’s disease were associated with more severe motor symptoms. Importantly, *SPP1*-null mice demonstrated reduced neurodegeneration [57]. Besides, SPP1 upregulated lysyl oxidase, an enzyme involved in cross-linking insoluble collagen in fibroblasts. An excess of SPP1 was associated with left-ventricular stiffness and systolic dysfunction in patients with chronic heart failure and hypertensive heart disease [58]. The levels of active TGF-beta and MMP-2, two essential fibrogenic signaling mediators, as well as type I collagen expression were significantly attenuated in *SPP1*-null mice treated with bleomycin, fibrosis inducer, compared to wild-type controls [59]. Taken together, these findings strongly suggest that suppression of SPP1 is a highly potential therapeutic approach for aging and AADs.

### ITGB5

Integrin alpha V beta 5 (ITGB5) encodes a subunit of integrin that can interact with several alpha chains to form a variety of integrin heterodimers. *ITGB5* was consistently upregulated in more than 60% comparisons in all four AAD classes, and the logFC of *ITGB5* in AADs was significantly higher than NAADs (*p* < 0.05, Figure 6C). Consistently, *ITGB5* was also found to be upregulated in chronic kidney disease and psoriatic arthritis [60, 61]. In particular, ITGB5 was significantly increased in the serum of patients with psoriatic arthritis, a distinct inflammatory arthritis occurring in 30% of psoriasis patients [60]. ITGB5 was suggested as a biomarker for both nonprogressive and progressive kidney diseases [61], and was also one of the genes strongly associated with ischemic heart disease [62]. Moreover, ITGB5 served as a ligand for Cyr61, a molecule stimulating the production of IL-6, which is considered an aging biomarker, via itgav/itgb5/Akt/NF-κB signaling pathway in RA [63, 64], further supporting its role in various mechanisms underlying multiple AADs.

### ADAMTS14

A disintegrin and metalloproteinase with thrombospondin motifs 14 (ADAMTS14) cleaves the amino-propeptides of fibrillar collagens, enabling collagen fibril formation prior to assembly of collagen, a major extracellular matrix (ECM) protein. *ADAMTS14 was* uniformly upregulated in more than 65% comparisons in neurological and fibrotic diseases. The logFC of *ADAMTS14* in AADs was also significantly higher than in NAADs (*p* < 0.01, Figure 6D). Significant upregulation of ADAMTS14 was observed in human osteoarthritis (OA) cartilage, suggesting its involvement in cartilage matrix anabolism [65]. ADAMTS14 was also linked to the susceptibility to aging-related Alzheimer's disease as well as the regulation of immune functions via TGF-beta signaling [66]. *ADAMTS14*-deficient mice remained healthy, fertile, and displayed normal amino-procollagen processing [67], suggesting that antagonizing ADAMTS14 is unlikely to result in severe toxicity. As such, pharmaceutical inhibition of ADAMTS14 may provide a promising therapeutic approach for aging and AADs.

## DISCUSSION

In recent years, extensive efforts have been applied to generating a wide range of transcriptomic, genomic, proteomic, imaging, methylation, and metagenomic aging-related data. However, analysis of such a massive amount of data requires tailored computational approaches, capable of providing a detailed overview of the aging process as well as identifying promising targets for delaying aging and treating age-associated diseases. PandaOmics has several unique advantages with respect to user experience, integrated deep learning-based algorithms, the comprehensive database, and the time machine validation approach [19]. In contrast to other alternatives, PandaOmics platform consists of a diverse set of validated AI analytical models (such as text mining, entity recognition, target ranking, and trend prediction), coupled with the ability to discover novel targets automatically, making this platform unique in the community.

While we acknowledge that inclusion and exclusion of AADs can impact the outcome of our analyses, unfortunately, we could not include all AADs into our study due to limited publicly available datasets for some diseases. Given this limitation, the selection of 14 AADs was based on the consideration of whether age is a strong risk factor for the disease’s onset, as well as the availability of public datasets. Cardiovascular diseases were not included in this study due to their common mechanistic root contributing to the insufficient blood supply to multiple organs [68, 69]. Cancers were also excluded, as some of the pathways and mechanisms implicated in tumorigenesis are contradictory to those typically implicated in aging, such as increased cell proliferation [70]. Regarding target selection, some of the aging-associated genes were filtered out due to target family consideration, for example, transcription factors and generic proteins were not included. Furthermore, the current analysis only retrieved transcriptomics data, which, in turn, restricted the depth of analysis. The incorporation of genomic data could bring deeper insights into the shared genetics between aging and aging-associated disorders. Moreover, as aimed to identify dual-purpose targets across aging and multiple AADs, genes that did not meet the dual-purpose were not selected by this approach. It is also worthy to note the trade-off between target novelty and the evidence connecting a target to a disease. The degree of novelty is defined by the volume of related publications, and thus increasing the novelty level will sacrifice the evidence supporting the target’s participation in the disease. Therefore, some of the highly novel targets selected by PandaOmics, were not mapped to any aging hallmarks due to the lack of literature support. Nevertheless, they could be potential aging-related target candidates worthy of further investigation.

By combining genes derived from a variety of AADs, we were able to establish potential targets at different levels of novelty. The subsequent association of these targets with pathways known to be involved in aging such as *MTOR*, *SIRT1*, *IGF1*, and *AKT1*. Interestingly, the well-known aging-related genes were often the top-ranked targets in both AADs and NAADs, possibly due to their involvement in a wide spectrum of pathways. Among high confidence targets with the most associated hallmarks were *IGF1R*, *HGF*, *IL6*, *MMP1*, *PARP1*, *SPP1*, and *ROCK1*. Whereas in terms of novel targets, we found *MYSM1*, *KAT6A*, *UBE2E3*, *RAB7B*, *RAB8B*, and *USP2*. The most frequently associated hallmark of our targets is inflammation. Each proposed target is associated with distinct patterns of aging hallmarks, suggesting complex mechanisms underlying the aging process. Nonetheless, targets associated with multiple hallmarks of aging should be considered for further studies. Notably, while some of the targets revealed by our analysis (such as *IGF1R, HGF, and KAT6A*), are well-characterized drivers of tumorigenesis, others are known tumor suppressors e.g., *PTEN*, *EP300*. While these targets may have a theoretic therapeutic potential in AADs setting, modulating these molecules may elevate the risk of cancer development, and they should be excluded from further consideration.

By further evaluating targets linked to the aging hallmarks and expression changes in AADs, 9 potential candidates were revealed. Many of these targets play roles in inflammation, which is in line with the view that inflammation is associated with multiple age-related diseases and is an intrinsic and major component of the aging processes [71]. As previous studies have also reported strong overlaps of inflammation-related genes between aging and age-related diseases [14], targeting the immune dysfunction in aging could be a powerful approach for improving healthspan [72]. In addition, several strong candidate targets play roles in ECM remodeling. While this signaling network is not a hallmark of aging, it clearly plays an important role during aging [73]. As such, our findings support the view that ECM can be considered as a hallmark of aging and a promising therapeutic target for developing interventions [9].

Considering the potential targets we selected in the present study, the clinical relevance of CXCL12, SPP1, ITGB5 and ADAMTS14 in neurodegenerative, auto-immune, and inflammatory conditions was demonstrated in AADs. Thus, targeting these genes may have major health and clinical benefits for both aging and AADs. Stromal aging fibroblasts expressed and secreted a higher level of CXCL12 than the young cell [74]. In addition, CXCL12 demonstrated an activating role on mature osteoclast by promoting bone-resorbing activity [75], supporting the observation that CXCL12 plasma level was inversely correlated with bone mineral density [76]. Consistently, with age, the rate of bone resorption exceeded that of bone formation, leading to bone loss. SPP1 modulated osteoclast differentiation [77], and its levels in the plasma of aged human donors were significantly higher than in young individuals, both in a normal state or upon muscle injury [78]. SPP1 was demonstrated to attenuate the regenerative responses of old muscle stem cells. Neutralization of SPP1 recovered and enhanced the myogenic responses of old muscle stem cells, but failed to induce significant effect in young muscle stem cells, revealing the inhibitory effects of the age-dependent increase in SPP1 level on skeletal muscle regeneration [79]. Besides the age-dependent inflammation and bone loss, with age, collagen fibers became fragmented and stiff [73], disrupting various aspects of homeostasis and affecting healthy function. For example, aged fibroblast-ECM interactions were disrupted due to the fragmentation of collagen fibrils. Such fibroblasts synthesized fewer ECM proteins and more matrix-degrading metalloproteinases [80]. ADAMTS14 participates in degradation of ECM collagen. Aging-related increase in ECM stiffness leads to an imbalance in matrix components as well as deposition and cross-linking of collagen [81]. Other than collagen, fibronectin is also a component of the ECM, where ITGAV:ITGB5 is one of the receptors for fibronectin. Aging is associated with increased stretching of fibronectin fibrils and ECM maturation. ITGB5 was reported as a putative physiologic activator of TGF-β, leading to activation of ECM-bound latent TGF-β1 by traction. Consistently, *ITGB5* knockout demonstrated the absence of TGF-β-related phenotype. The most putative TGF-β activators are functionally associated with the ECM [82]. TGF-β signaling, being downstream of other signals, was shown to repress body size as well as lifespan *in vivo* [83]. Notably, ITGB5 knockdown did not affect the proliferation of human adipose-derived stem cells [84], suggesting minimal cell toxicity induced. Therefore, suppressing ITGB5 may provide new insights for aging treatment. Taken together, inhibition of CXCL12, SPP1, ITGB5 and ADAMTS14 may provide a promising therapeutic approach for aging and AADs.

Epigenetic reprogramming is one of the most promising areas in longevity [85]. In line with this notion, 13 of our targets (*HDAC1, HDAC9, EP300, KAT6A, KAT8, KDM7A, EZH2, DNMT1, SIRT1, MTOR, IGF1, AKT1*, and *MYO1C*) were associated with the epigenetic shift aging hallmark (Figure 3). Histone modification and DNA methylation are the most studied epigenetic phenomena, and these modifications are accumulating over the life course. Histone deacetylases HDAC1 and HDAC9 are markers of epigenetic transcriptional repression. Whereas histone acetyltransferases EP300, KAT6A, and KAT8 enhance epigenetic transcriptional activation [86–89]. Histone demethylase KDM7A specifically demethylates H3K9me2, H3K27me2 and H4K20me1. Histone-lysine N-methyltransferase EZH2 methylates H3K9me and H3K27me, leading to epigenetic transcriptional repression of the affected gene. Some of these epigenetic regulatory enzyme targets were also involved in modulating aging processes. For example, it was reported that KAT8 might alter the function of ATM, which plays a pro-longevity role [90, 91]. In addition, the activities of DNMT1 and SIRT1 were found to be attenuated during aging, leading to alterations of epigenetic landscape, thereby changing gene expression and promoting aging processes [3]. In aging livers, C/EBPβ-HDAC1 complexes repress E2F-dependent promoters and occupy the promoter of GSK3B, resulting in epigenetic silencing of cell cycle genes and altered GSKβ-cyclin D3 pathways, respectively [92]. Collectively, the above evidence reveals the involvement of our target in epigenetic regulation of cellular proliferation and development, and suggests the potential mechanism for the involvement of these targets in aging and AADs. Targeting epigenetic regulation may be one of the promising approaches for healthspan-promotion and life-extension [93].

In conclusion, we successfully established an approach to identify potential dual-purpose targets for aging and AADs, enabling biologists and clinicians to further investigate their therapeutic potential in a cost-saving and time-efficient manner for drug discovery. These promising results underscore the ability of PandaOmics to identify novel targets not only for specific disorders, but across multiple types of diseases.

## MATERIALS AND METHODS

### Disease and dataset selection

Diseases were selected and classified into either AADs or NAADs based on the consideration of whether age is a strong risk factor for the disease’s onset. To obtain a more aging-oriented result, 14 AADs and 19 NAADs were selected (Table 1).

Microarray and RNA-seq datasets for the selected diseases containing case and control samples were retrieved from public repositories and processed by PandaOmics (Supplementary Table 7). A total of 79 and 113 datasets were selected for AADs and NAADs, respectively. For AADs, age information was available in 29 datasets, with 1,223 cases and 819 control samples. For NAADs, age information was available in 35 datasets containing 1,161 cases and 713 control samples. The mean age of cases and controls in AADs was 67.9 (s.d. = 17.50) and 60.91 (s.d. = 21.01), and in NAADs 36.87 (s.d. = 18.29) and 37.20 (s.d. = 19.15), respectively.

### Meta-analysis

For each dataset, case and control samples from the same tissue source were selected and compared, resulting in a total of 87 and 126 case-control comparisons for 14 AADs and 19 NAADs, respectively (Table 1). All the case-control comparisons performed for each disease were pooled into a single meta-analysis, yielding a total of 33 meta-analyses for all selected diseases subjected to target identification.

### Filter settings for target identification

Targets were prioritized by PandaOmics (available at https://pandaomics.com/) using its AI hypothesis generation models based on 21 scores from Omics, Text-based, Financial, and KOL categories. Additional filters including Druggability (small molecules, antibodies, safety, novelty), Tissue specificity, Target family, and Development filters were applied to refine the list to satisfy the user’s research goals. In this study, only the genes belonging to the druggable protein class were included. The loss of novelty would be a trade-off for the abundance of evidence connecting a target to a disease. In view of this, a list of target genes in high confidence, medium novelty, and high novelty settings based on the volume of related publications proposed by PandaOmics’ proprietary AI engine, as well as the number of clinical trials they have been involved in was identified. A customized set of scores and filters was applied to obtain a list of genes with the associated final ranking, which represents the strength of association between a gene and the disease, for each novelty setting.

### Identification of targets implicated in multiple diseases

For each novelty setting, a list of 100 genes with the highest ranking calculated by PandaOmics was extracted from each disease, generating a combined list of genes from 14 AADs, and another from 19 NAADs. The genes were then prioritized by their (1) descending occurrence, and (2) ascending average ranking across multiple diseases, and those top-100 genes were selected for further analysis. Consequently, these selected genes from AADs were overlapped with those from NAADs to classify the genes into AAD targets and common targets, as exemplified in the Venn diagram (Figure 1).

### Hallmarks of aging assessment

The 300 genes obtained from the three novelty settings consisting of both AAD targets and common targets were subjected to literature review on PubMed for their association with hallmarks of aging (search terms included in Supplementary Table 2). Studies that matched our search terms composed of all hallmarks and keywords of the corresponding pathways were selected for review. Their association with hallmarks of aging was decided based on their biological functions, pathways, and roles in regulating important pathways or genes associated with aging. All genes associated with hallmarks of aging were included, along with their literature evidence and PubMed ID (Supplementary Table 2). Among the genes included, those that are known cancer drivers were annotated by the data of the NCG7.0 database [33].

### Expression levels in age-associated diseases

The values of logFC for the genes in each of the 87 case-control comparisons performed for AADs were calculated. Considering the diverse complexity of mechanisms and pathologies in different diseases, we computed the consistency of each gene’s dysregulation state in each of the four disease classes (fibrotic, inflammatory, metabolic, and neurological diseases). Genes that were upregulated or downregulated in 60% or more of case-control comparisons in the disease class were considered as consistently dysregulated. Genes were further investigated provided that they were consistently dysregulated in the same trend in 2 or more disease classes.

### Pathway enrichment analysis for identified targets

The 145 genes identified from hallmarks of aging assessment were input to perform pathway enrichment analysis based on the KEGG PATHWAY Database [48] by *clusterProfiler* in R. Pathways with *p* < 0.05 were considered significantly enriched. Aging-associated pathways were further selected for visualization.

### Curation of known aging-associated genes

The curation of well-known genes associated with aging was based on the genes targeted by the investigated drugs that entered clinical trials with either aging or healthy aging as one of the disease conditions (http://ClinicalTrials.gov, accessed on 30-DEC-2021), publication and geroprotectors (http://Geroprotectors.org, accessed on 17-FEB-2022) [46]. The curated genes were further refined to druggable genes in PandaOmics by (1) applying the filter of a druggable protein class and (2) adjusting the druggability filter (small molecule score ≥1 and safety score ≥1), yielding the final list of 62, 48 and 52 known aging-associated genes from clinical trials (Supplementary Table 3), publication (Supplementary Table 4) and geroprotectors (Supplementary Table 5), respectively. In addition, 307 genes associated with aging suggested by the database, GenAge (build 20) [47], were also retrieved and filtered based on the above settings, resulting in 149 genes included for analysis.

### Statistical analysis

*T*-test analysis was performed to compare the logFC (two-tailed) for each gene calculated by PandaOmics between AADs and NAADs. The significant level of target enrichment in the pool of curated aging-associated genes or GenAge genes was estimated using the hypergeometric test as:


$$p=1-\sum_{i=0}^{r-1}\frac{\left(\frac{K}{i}\right)\left(\frac{N-K}{n-i}\right)}{\left(\frac{N}{n}\right)}$$

where *N* equals 5,626 which stands for the total number of druggable genes defined in PandaOmics, *K* represents the number of aging-associated genes in the interested pool, *n* is the number of identified targets, and *r* represents the number of genes shared between the interested pool of aging-associated genes and the list of identified targets.

## Supplementary Materials

Supplementary Figures

Supplementary Tables 1-2 and 6-7

Supplementary Tables 3-5 and 8

## References

[1] de Magalhães JP, Wuttke D, Wood SH, Plank M, Vora C. Genome-environment interactions that modulate aging: powerful targets for drug discovery. Pharmacol Rev. 2012; 64:88–101. 10.1124/pr.110.004499 22090473 PMC3250080

[2] Kennedy BK, Berger SL, Brunet A, Campisi J, Cuervo AM, Epel ES, Franceschi C, Lithgow GJ, Morimoto RI, Pessin JE, Rando TA, Richardson A, Schadt EE, et al. Geroscience: linking aging to chronic disease. Cell. 2014; 159:709–13. 10.1016/j.cell.2014.10.039 25417146 PMC4852871

[3] Moskalev AA, Aliper AM, Smit-McBride Z, Buzdin A, Zhavoronkov A. Genetics and epigenetics of aging and longevity. Cell Cycle. 2014; 13:1063–77. 10.4161/cc.28433 24603410 PMC4013158

[4] Moskalev A, Chernyagina E, Tsvetkov V, Fedintsev A, Shaposhnikov M, Krut’ko V, Zhavoronkov A, Kennedy BK. Developing criteria for evaluation of geroprotectors as a key stage toward translation to the clinic. Aging Cell. 2016; 15:407–15. 10.1111/acel.12463 26970234 PMC4854916

[5] de Magalhães JP. Longevity pharmacology comes of age. Drug Discov Today. 2021; 26:1559–62. 10.1016/j.drudis.2021.02.015 33617794

[6] Partridge L, Fuentealba M, Kennedy BK. The quest to slow ageing through drug discovery. Nat Rev Drug Discov. 2020; 19:513–32. 10.1038/s41573-020-0067-7 32467649

[7] Moskalev A, Guvatova Z, Lopes IA, Beckett CW, Kennedy BK, De Magalhaes JP, Makarov AA. Targeting aging mechanisms: pharmacological perspectives. Trends Endocrinol Metab. 2022; 33:266–80. 10.1016/j.tem.2022.01.007 35183431

[8] López-Otín C, Blasco MA, Partridge L, Serrano M, Kroemer G. The hallmarks of aging. Cell. 2013; 153:1194–217. 10.1016/j.cell.2013.05.039 23746838 PMC3836174

[9] Fedintsev A, Moskalev A. Stochastic non-enzymatic modification of long-lived macromolecules - A missing hallmark of aging. Ageing Res Rev. 2020; 62:101097. 10.1016/j.arr.2020.101097 32540391

[10] Gorbunova V, Seluanov A, Mita P, McKerrow W, Fenyö D, Boeke JD, Linker SB, Gage FH, Kreiling JA, Petrashen AP, Woodham TA, Taylor JR, Helfand SL, Sedivy JM. The role of retrotransposable elements in ageing and age-associated diseases. Nature. 2021; 596:43–53. 10.1038/s41586-021-03542-y 34349292 PMC8600649

[11] Franceschi C, Garagnani P, Parini P, Giuliani C, Santoro A. Inflammaging: a new immune-metabolic viewpoint for age-related diseases. Nat Rev Endocrinol. 2018; 14:576–90. 10.1038/s41574-018-0059-4 30046148

[12] Childs BG, Durik M, Baker DJ, van Deursen JM. Cellular senescence in aging and age-related disease: from mechanisms to therapy. Nat Med. 2015; 21:1424–35. 10.1038/nm.4000 26646499 PMC4748967

[13] Gasek NS, Kuchel GA, Kirkland JL, Xu M. Strategies for Targeting Senescent Cells in Human Disease. Nat Aging. 2021; 1:870–9. 10.1038/s43587-021-00121-8 34841261 PMC8612694

[14] Fernandes M, Wan C, Tacutu R, Barardo D, Rajput A, Wang J, Thoppil H, Thornton D, Yang C, Freitas A, de Magalhães JP. Systematic analysis of the gerontome reveals links between aging and age-related diseases. Hum Mol Genet. 2016; 25:4804–18. 10.1093/hmg/ddw307 28175300 PMC5418736

[15] Dugani SB, Hydoub YM, Ayala AP, Reka R, Nayfeh T, Ding JF, McCafferty SN, Alzuabi M, Farwati M, Murad MH, Alsheikh-Ali AA, Mora S. Risk Factors for Premature Myocardial Infarction: A Systematic Review and Meta-analysis of 77 Studies. Mayo Clin Proc Innov Qual Outcomes. 2021; 5:783–94. 10.1016/j.mayocpiqo.2021.03.009 34401655 PMC8358212

[16] Zhavoronkov A. Geroprotective and senoremediative strategies to reduce the comorbidity, infection rates, severity, and lethality in gerophilic and gerolavic infections. Aging (Albany NY). 2020; 12:6492–510. 10.18632/aging.102988 32229705 PMC7202545

[17] Paul SM, Mytelka DS, Dunwiddie CT, Persinger CC, Munos BH, Lindborg SR, Schacht AL. How to improve R&D productivity: the pharmaceutical industry's grand challenge. Nat Rev Drug Discov. 2010; 9:203–14. 10.1038/nrd3078 20168317

[18] Arrowsmith J. Trial watch: Phase II failures: 2008-2010. Nat Rev Drug Discov. 2011; 10:328–9. 10.1038/nrd3439 21532551

[19] Zhavoronkov A, Mamoshina P, Vanhaelen Q, Scheibye-Knudsen M, Moskalev A, Aliper A. Artificial intelligence for aging and longevity research: Recent advances and perspectives. Ageing Res Rev. 2019; 49:49–66. 10.1016/j.arr.2018.11.003 30472217

[20] Vega Magdaleno GD, Bespalov V, Zheng Y, Freitas AA, de Magalhaes JP. Machine learning-based predictions of dietary restriction associations across ageing-related genes. BMC Bioinformatics. 2022; 23:10. 10.1186/s12859-021-04523-8 34983372 PMC8729156

[21] Fabris F, Palmer D, Salama KM, de Magalhães JP, Freitas AA. Using deep learning to associate human genes with age-related diseases. Bioinformatics. 2020; 36:2202–8. 10.1093/bioinformatics/btz887 31845988 PMC7141856

[22] Barardo DG, Newby D, Thornton D, Ghafourian T, de Magalhães JP, Freitas AA. Machine learning for predicting lifespan-extending chemical compounds. Aging (Albany NY). 2017; 9:1721–37. 10.18632/aging.101264 28783712 PMC5559171

[23] Galkin F, Mamoshina P, Aliper A, de Magalhães JP, Gladyshev VN, Zhavoronkov A. Biohorology and biomarkers of aging: Current state-of-the-art, challenges and opportunities. Ageing Res Rev. 2020; 60:101050. 10.1016/j.arr.2020.101050 32272169

[24] Zhavoronkov A, Bischof E, Lee KF. Artificial intelligence in longevity medicine. Nat Aging. 2021; 1:5–7. 10.1038/s43587-020-00020-4 37118000

[25] Ozerov IV, Lezhnina KV, Izumchenko E, Artemov AV, Medintsev S, Vanhaelen Q, Aliper A, Vijg J, Osipov AN, Labat I, West MD, Buzdin A, Cantor CR, et al. In silico Pathway Activation Network Decomposition Analysis (iPANDA) as a method for biomarker development. Nat Commun. 2016; 7:13427. 10.1038/ncomms13427 27848968 PMC5116087

[26] West MD, Labat I, Sternberg H, Larocca D, Nasonkin I, Chapman KB, Singh R, Makarev E, Aliper A, Kazennov A, Alekseenko A, Shuvalov N, Cheskidova E, et al. Use of deep neural network ensembles to identify embryonic-fetal transition markers: repression of *COX7A1* in embryonic and cancer cells. Oncotarget. 2017; 9:7796–811. 10.18632/oncotarget.23748 29487692 PMC5814259

[27] Aliper A, Plis S, Artemov A, Ulloa A, Mamoshina P, Zhavoronkov A. Deep Learning Applications for Predicting Pharmacological Properties of Drugs and Drug Repurposing Using Transcriptomic Data. Mol Pharm. 2016; 13:2524–30. 10.1021/acs.molpharmaceut.6b00248 27200455 PMC4965264

[28] Aliper A, Belikov AV, Garazha A, Jellen L, Artemov A, Suntsova M, Ivanova A, Venkova L, Borisov N, Buzdin A, Mamoshina P, Putin E, Swick AG, et al. In search for geroprotectors: in silico screening and in vitro validation of signalome-level mimetics of young healthy state. Aging (Albany NY). 2016; 8:2127–52. 10.18632/aging.101047 27677171 PMC5076455

[29] Aliper A, Jellen L, Cortese F, Artemov A, Karpinsky-Semper D, Moskalev A, Swick AG, Zhavoronkov A. Towards natural mimetics of metformin and rapamycin. Aging (Albany NY). 2017; 9:2245–68. 10.18632/aging.101319 29165314 PMC5723685

[30] Hale C. Breaking Big Pharma's AI barrier: Insilico Medicine uncovers novel target, new drug for pulmonary fibrosis in 18 months. Fierce Biotech: USA; 2021.

[31] Hale C. Insilico Medicine begins first human trial of its AI-designed drug for pulmonary fibrosis. Fierce Biotech: USA; 2021.

[32] Hale C. Insilico Medicine's AI engines continue to churn out new drug candidates, now in kidney fibrosis. Fierce Biotech: USA; 2021.

[33] Dressler L, Bortolomeazzi M, Keddar MR, Misetic H, Sartini G, Acha-Sagredo A, Montorsi L, Wijewardhane N, Repana D, Nulsen J, Goldman J, Pollitt M, Davis P, et al. Comparative assessment of genes driving cancer and somatic evolution in non-cancer tissues: an update of the Network of Cancer Genes (NCG) resource. Genome Biol. 2022; 23:35. 10.1186/s13059-022-02607-z 35078504 PMC8790917

[34] Longo VD, Kennedy BK. Sirtuins in aging and age-related disease. Cell. 2006; 126:257–68. 10.1016/j.cell.2006.07.002 16873059

[35] Elibol B, Kilic U. High Levels of SIRT1 Expression as a Protective Mechanism Against Disease-Related Conditions. Front Endocrinol (Lausanne). 2018; 9:614. 10.3389/fendo.2018.00614 30374331 PMC6196295

[36] Blagosklonny MV. An anti-aging drug today: from senescence-promoting genes to anti-aging pill. Drug Discov Today. 2007; 12:218–24. 10.1016/j.drudis.2007.01.004 17331886

[37] Li J, Kim SG, Blenis J. Rapamycin: one drug, many effects. Cell Metab. 2014; 19:373–9. 10.1016/j.cmet.2014.01.001 24508508 PMC3972801

[38] Papadopoli D, Boulay K, Kazak L, Pollak M, Mallette F, Topisirovic I, Hulea L. mTOR as a central regulator of lifespan and aging. F1000Res. 2019; 8:998. 10.12688/f1000research.17196.1 31316753 PMC6611156

[39] Arum O, Boparai RK, Saleh JK, Wang F, Dirks AL, Turner JG, Kopchick JJ, Liu JL, Khardori RK, Bartke A. Specific suppression of insulin sensitivity in growth hormone receptor gene-disrupted (GHR-KO) mice attenuates phenotypic features of slow aging. Aging Cell. 2014; 13:981–1000. 10.1111/acel.12262 25244225 PMC4326932

[40] Palacios JA, Herranz D, De Bonis ML, Velasco S, Serrano M, Blasco MA. SIRT1 contributes to telomere maintenance and augments global homologous recombination. J Cell Biol. 2010; 191:1299–313. 10.1083/jcb.201005160 21187328 PMC3010065

[41] Osum M, Serakinci N. Impact of circadian disruption on health; SIRT1 and Telomeres. DNA Repair (Amst). 2020; 96:102993. 10.1016/j.dnarep.2020.102993 33038659

[42] Meng T, Qin W, Liu B. SIRT1 Antagonizes Oxidative Stress in Diabetic Vascular Complication. Front Endocrinol (Lausanne). 2020; 11:568861. 10.3389/fendo.2020.568861 33304318 PMC7701141

[43] Kim TH, Yang YM, Han CY, Koo JH, Oh H, Kim SS, You BH, Choi YH, Park TS, Lee CH, Kurose H, Noureddin M, Seki E, et al. Gα12 ablation exacerbates liver steatosis and obesity by suppressing USP22/SIRT1-regulated mitochondrial respiration. J Clin Invest. 2018; 128:5587–602. 10.1172/JCI97831 30300140 PMC6264648

[44] Yang YL, Wang PW, Wang FS, Lin HY, Huang YH. miR-29a Modulates GSK3β/SIRT1-Linked Mitochondrial Proteostatic Stress to Ameliorate Mouse Non-Alcoholic Steatohepatitis. Int J Mol Sci. 2020; 21:6884. 10.3390/ijms21186884 32961796 PMC7555728

[45] Lin YF, Sam J, Evans T. Sirt1 promotes tissue regeneration in zebrafish through regulating the mitochondrial unfolded protein response. iScience. 2021; 24:103118. 10.1016/j.isci.2021.103118 34622167 PMC8479786

[46] Moskalev A, Chernyagina E, de Magalhães JP, Barardo D, Thoppil H, Shaposhnikov M, Budovsky A, Fraifeld VE, Garazha A, Tsvetkov V, Bronovitsky E, Bogomolov V, Scerbacov A, et al. Geroprotectors.org: a new, structured and curated database of current therapeutic interventions in aging and age-related disease. Aging (Albany NY). 2015; 7:616–28. 10.18632/aging.100799 26342919 PMC4600621

[47] Tacutu R, Thornton D, Johnson E, Budovsky A, Barardo D, Craig T, Diana E, Lehmann G, Toren D, Wang J, Fraifeld VE, de Magalhães JP. Human Ageing Genomic Resources: new and updated databases. Nucleic Acids Res. 2018; 46:D1083–90. 10.1093/nar/gkx1042 29121237 PMC5753192

[48] Kanehisa M, Goto S. KEGG: kyoto encyclopedia of genes and genomes. Nucleic Acids Res. 2000; 28:27–30. 10.1093/nar/28.1.27 10592173 PMC102409

[49] Gilbert W, Bragg R, Elmansi AM, McGee-Lawrence ME, Isales CM, Hamrick MW, Hill WD, Fulzele S. Stromal cell-derived factor-1 (CXCL12) and its role in bone and muscle biology. Cytokine. 2019; 123:154783. 10.1016/j.cyto.2019.154783 31336263 PMC6948927

[50] Zhang Y, Dépond M, He L, Foudi A, Kwarteng EO, Lauret E, Plo I, Desterke C, Dessen P, Fujii N, Opolon P, Herault O, Solary E, et al. CXCR4/CXCL12 axis counteracts hematopoietic stem cell exhaustion through selective protection against oxidative stress. Sci Rep. 2016; 6:37827. 10.1038/srep37827 27886253 PMC5122894

[51] Li F, Xu X, Geng J, Wan X, Dai H. The autocrine CXCR4/CXCL12 axis contributes to lung fibrosis through modulation of lung fibroblast activity. Exp Ther Med. 2020; 19:1844–54. 10.3892/etm.2020.8433 32104240 PMC7027131

[52] Gao B, Sun G, Wang Y, Geng Y, Zhou L, Chen X. microRNA-23 inhibits inflammation to alleviate rheumatoid arthritis via regulating CXCL12. Exp Ther Med. 2021; 21:459. 10.3892/etm.2021.9890 33777193 PMC7967800

[53] Andrés-Benito P, Povedano M, Domínguez R, Marco C, Colomina MJ, López-Pérez Ó, Santana I, Baldeiras I, Martínez-Yelámos S, Zerr I, Llorens F, Fernández-Irigoyen J, Santamaría E, Ferrer I. Increased C-X-C Motif Chemokine Ligand 12 Levels in Cerebrospinal Fluid as a Candidate Biomarker in Sporadic Amyotrophic Lateral Sclerosis. Int J Mol Sci. 2020; 21:8680. 10.3390/ijms21228680 33213069 PMC7698527

[54] Rabinovich-Nikitin I, Ezra A, Barbiro B, Rabinovich-Toidman P, Solomon B. Chronic administration of AMD3100 increases survival and alleviates pathology in SOD1(G93A) mice model of ALS. J Neuroinflammation. 2016; 13:123. 10.1186/s12974-016-0587-6 27230771 PMC4882847

[55] Zhao H, Chen Q, Alam A, Cui J, Suen KC, Soo AP, Eguchi S, Gu J, Ma D. The role of osteopontin in the progression of solid organ tumour. Cell Death Dis. 2018; 9:356. 10.1038/s41419-018-0391-6 29500465 PMC5834520

[56] Morisaki Y, Niikura M, Watanabe M, Onishi K, Tanabe S, Moriwaki Y, Okuda T, Ohara S, Murayama S, Takao M, Uchida S, Yamanaka K, Misawa H. Selective Expression of Osteopontin in ALS-resistant Motor Neurons is a Critical Determinant of Late Phase Neurodegeneration Mediated by Matrix Metalloproteinase-9. Sci Rep. 2016; 6:27354. 10.1038/srep27354 27264390 PMC4893611

[57] Maetzler W, Berg D, Schalamberidze N, Melms A, Schott K, Mueller JC, Liaw L, Gasser T, Nitsch C. Osteopontin is elevated in Parkinson's disease and its absence leads to reduced neurodegeneration in the MPTP model. Neurobiol Dis. 2007; 25:473–82. 10.1016/j.nbd.2006.10.020 17188882

[58] López B, González A, Lindner D, Westermann D, Ravassa S, Beaumont J, Gallego I, Zudaire A, Brugnolaro C, Querejeta R, Larman M, Tschöpe C, Díez J. Osteopontin-mediated myocardial fibrosis in heart failure: a role for lysyl oxidase? Cardiovasc Res. 2013; 99:111–20. 10.1093/cvr/cvt100 23619422

[59] Berman JS, Serlin D, Li X, Whitley G, Hayes J, Rishikof DC, Ricupero DA, Liaw L, Goetschkes M, O’Regan AW. Altered bleomycin-induced lung fibrosis in osteopontin-deficient mice. Am J Physiol Lung Cell Mol Physiol. 2004; 286:L1311–8. 10.1152/ajplung.00394.2003 14977630

[60] Cretu D, Liang K, Saraon P, Batruch I, Diamandis EP, Chandran V. Quantitative tandem mass-spectrometry of skin tissue reveals putative psoriatic arthritis biomarkers. Clin Proteomics. 2015; 12:1. 10.1186/1559-0275-12-1 25678896 PMC4304122

[61] Ju W, Eichinger F, Bitzer M, Oh J, McWeeney S, Berthier CC, Shedden K, Cohen CD, Henger A, Krick S, Kopp JB, Stoeckert CJ Jr, Dikman S, et al. Renal gene and protein expression signatures for prediction of kidney disease progression. Am J Pathol. 2009; 174:2073–85. 10.2353/ajpath.2009.080888 19465643 PMC2684173

[62] Schooling CM, Huang JV, Zhao JV, Kwok MK, Au Yeung SL, Lin SL. Disconnect Between Genes Associated With Ischemic Heart Disease and Targets of Ischemic Heart Disease Treatments. EBioMedicine. 2018; 28:311–5. 10.1016/j.ebiom.2018.01.015 29396305 PMC5835561

[63] Johnson TE. Recent results: biomarkers of aging. Exp Gerontol. 2006; 41:1243–6. 10.1016/j.exger.2006.09.006 17071038

[64] Lin J, Zhou Z, Huo R, Xiao L, Ouyang G, Wang L, Sun Y, Shen B, Li D, Li N. Cyr61 induces IL-6 production by fibroblast-like synoviocytes promoting Th17 differentiation in rheumatoid arthritis. J Immunol. 2012; 188:5776–84. 10.4049/jimmunol.1103201 22547695

[65] Yang CY, Chanalaris A, Troeberg L. ADAMTS and ADAM metalloproteinases in osteoarthritis - looking beyond the 'usual suspects'. Osteoarthritis Cartilage. 2017; 25:1000–9. 10.1016/j.joca.2017.02.791 28216310 PMC5473942

[66] Mukherjee S, Heath L, Preuss C, Jayadev S, Garden GA, Greenwood AK, Sieberts SK, De Jager PL, Ertekin-Taner N, Carter GW, Mangravite LM, Logsdon BA. Molecular estimation of neurodegeneration pseudotime in older brains. Nat Commun. 2020; 11:5781. 10.1038/s41467-020-19622-y 33188183 PMC7666177

[67] Dupont L, Ehx G, Chantry M, Monseur C, Leduc C, Janssen L, Cataldo D, Thiry M, Jerome C, Thomassin JM, Nusgens B, Dubail J, Baron F, Colige A. Spontaneous atopic dermatitis due to immune dysregulation in mice lacking Adamts2 and 14. Matrix Biol. 2018; 70:140–57. 10.1016/j.matbio.2018.04.002 29649548

[68] Dutta P, Courties G, Wei Y, Leuschner F, Gorbatov R, Robbins CS, Iwamoto Y, Thompson B, Carlson AL, Heidt T, Majmudar MD, Lasitschka F, Etzrodt M, et al. Myocardial infarction accelerates atherosclerosis. Nature. 2012; 487:325–9. 10.1038/nature11260 22763456 PMC3401326

[69] Ross R. Atherosclerosis--an inflammatory disease. N Engl J Med. 1999; 340:115–26. 10.1056/NEJM199901143400207 9887164

[70] Chatsirisupachai K, Palmer D, Ferreira S, de Magalhães JP. A human tissue-specific transcriptomic analysis reveals a complex relationship between aging, cancer, and cellular senescence. Aging Cell. 2019; 18:e13041. 10.1111/acel.13041 31560156 PMC6826163

[71] Ferrucci L, Fabbri E. Inflammageing: chronic inflammation in ageing, cardiovascular disease, and frailty. Nat Rev Cardiol. 2018; 15:505–22. 10.1038/s41569-018-0064-2 30065258 PMC6146930

[72] Borgoni S, Kudryashova KS, Burka K, de Magalhães JP. Targeting immune dysfunction in aging. Ageing Res Rev. 2021; 70:101410. 10.1016/j.arr.2021.101410 34280555

[73] Birch HL. Extracellular Matrix and Ageing. Subcell Biochem. 2018; 90:169–90. 10.1007/978-981-13-2835-0_7 30779010

[74] Begley L, Monteleon C, Shah RB, Macdonald JW, Macoska JA. CXCL12 overexpression and secretion by aging fibroblasts enhance human prostate epithelial proliferation in vitro. Aging Cell. 2005; 4:291–8. 10.1111/j.1474-9726.2005.00173.x 16300481

[75] Grassi F, Cristino S, Toneguzzi S, Piacentini A, Facchini A, Lisignoli G. CXCL12 chemokine up-regulates bone resorption and MMP-9 release by human osteoclasts: CXCL12 levels are increased in synovial and bone tissue of rheumatoid arthritis patients. J Cell Physiol. 2004; 199:244–51. 10.1002/jcp.10445 15040007

[76] Carbone LD, Bůžková P, Fink HA, Robbins JA, Bethel M, Hamrick MW, Hill WD. Association of Plasma SDF-1 with Bone Mineral Density, Body Composition, and Hip Fractures in Older Adults: The Cardiovascular Health Study. Calcif Tissue Int. 2017; 100:599–608. 10.1007/s00223-017-0245-8 28246930 PMC5649737

[77] Rittling SR, Matsumoto HN, McKee MD, Nanci A, An XR, Novick KE, Kowalski AJ, Noda M, Denhardt DT. Mice lacking osteopontin show normal development and bone structure but display altered osteoclast formation in vitro. J Bone Miner Res. 1998; 13:1101–11. 10.1359/jbmr.1998.13.7.1101 9661074

[78] Lekwuwa M, Choudhary M, Lad EM, Malek G. Osteopontin accumulates in basal deposits of human eyes with age-related macular degeneration and may serve as a biomarker of aging. Mod Pathol. 2022; 35:165–76. 10.1038/s41379-021-00887-7 34389792 PMC8786662

[79] Paliwal P, Pishesha N, Wijaya D, Conboy IM. Age dependent increase in the levels of osteopontin inhibits skeletal muscle regeneration. Aging (Albany NY). 2012; 4:553–66. 10.18632/aging.100477 22915705 PMC3461343

[80] Cole MA, Quan T, Voorhees JJ, Fisher GJ. Extracellular matrix regulation of fibroblast function: redefining our perspective on skin aging. J Cell Commun Signal. 2018; 12:35–43. 10.1007/s12079-018-0459-1 29455303 PMC5842211

[81] Deasy SK, Erez N. A glitch in the matrix: organ-specific matrisomes in metastatic niches. Trends Cell Biol. 2022; 32:110–23. 10.1016/j.tcb.2021.08.001 34479765

[82] Munger JS, Sheppard D. Cross talk among TGF-β signaling pathways, integrins, and the extracellular matrix. Cold Spring Harb Perspect Biol. 2011; 3:a005017. 10.1101/cshperspect.a005017 21900405 PMC3220354

[83] Hirose T, Nakano Y, Nagamatsu Y, Misumi T, Ohta H, Ohshima Y. Cyclic GMP-dependent protein kinase EGL-4 controls body size and lifespan in C elegans. Development. 2003; 130:1089–99. 10.1242/dev.00330 12571101

[84] Morandi EM, Verstappen R, Zwierzina ME, Geley S, Pierer G, Ploner C. ITGAV and ITGA5 diversely regulate proliferation and adipogenic differentiation of human adipose derived stem cells. Sci Rep. 2016; 6:28889. 10.1038/srep28889 27363302 PMC4929468

[85] de Magalhães JP, Ocampo A. Cellular reprogramming and the rise of rejuvenation biotech. Trends Biotechnol. 2022. [Epub ahead print]. 10.1016/j.tibtech.2022.01.011 35190201

[86] Tropberger P, Pott S, Keller C, Kamieniarz-Gdula K, Caron M, Richter F, Li G, Mittler G, Liu ET, Bühler M, Margueron R, Schneider R. Regulation of transcription through acetylation of H3K122 on the lateral surface of the histone octamer. Cell. 2013; 152:859–72. 10.1016/j.cell.2013.01.032 23415232

[87] Delvecchio M, Gaucher J, Aguilar-Gurrieri C, Ortega E, Panne D. Structure of the p300 catalytic core and implications for chromatin targeting and HAT regulation. Nat Struct Mol Biol. 2013; 20:1040–6. 10.1038/nsmb.2642 23934153

[88] Ogryzko VV, Schiltz RL, Russanova V, Howard BH, Nakatani Y. The transcriptional coactivators p300 and CBP are histone acetyltransferases. Cell. 1996; 87:953–9. 10.1016/s0092-8674(00)82001-2 8945521

[89] Yuan H, Rossetto D, Mellert H, Dang W, Srinivasan M, Johnson J, Hodawadekar S, Ding EC, Speicher K, Abshiru N, Perry R, Wu J, Yang C, et al. MYST protein acetyltransferase activity requires active site lysine autoacetylation. EMBO J. 2012; 31:58–70. 10.1038/emboj.2011.382 22020126 PMC3252582

[90] Gupta A, Sharma GG, Young CS, Agarwal M, Smith ER, Paull TT, Lucchesi JC, Khanna KK, Ludwig T, Pandita TK. Involvement of human MOF in ATM function. Mol Cell Biol. 2005; 25:5292–305. 10.1128/MCB.25.12.5292-5305.2005 15923642 PMC1140595

[91] Qian M, Liu Z, Peng L, Tang X, Meng F, Ao Y, Zhou M, Wang M, Cao X, Qin B, Wang Z, Zhou Z, Wang G, et al. Boosting ATM activity alleviates aging and extends lifespan in a mouse model of progeria. Elife. 2018; 7:e34836. 10.7554/eLife.34836 29717979 PMC5957528

[92] Jin J, Wang GL, Shi X, Darlington GJ, Timchenko NA. The age-associated decline of glycogen synthase kinase 3beta plays a critical role in the inhibition of liver regeneration. Mol Cell Biol. 2009; 29:3867–80. 10.1128/MCB.00456-09 19398579 PMC2704742

[93] Pasyukova EG, Vaiserman AM. HDAC inhibitors: A new promising drug class in anti-aging research. Mech Ageing Dev. 2017; 166:6–15. 10.1016/j.mad.2017.08.008 28843433

[94] Tzeng YS, Li H, Kang YL, Chen WC, Cheng WC, Lai DM. Loss of Cxcl12/Sdf-1 in adult mice decreases the quiescent state of hematopoietic stem/progenitor cells and alters the pattern of hematopoietic regeneration after myelosuppression. Blood. 2011; 117:429–39. 10.1182/blood-2010-01-266833 20833981

[95] Guidi N, Sacma M, Ständker L, Soller K, Marka G, Eiwen K, Weiss JM, Kirchhoff F, Weil T, Cancelas JA, Florian MC, Geiger H. Osteopontin attenuates aging-associated phenotypes of hematopoietic stem cells. EMBO J. 2017; 36:840–53. 10.15252/embj.201694969 28254837 PMC5376966

[96] Hashimoto M, Sun D, Rittling SR, Denhardt DT, Young W. Osteopontin-deficient mice exhibit less inflammation, greater tissue damage, and impaired locomotor recovery from spinal cord injury compared with wild-type controls. J Neurosci. 2007; 27:3603–11. 10.1523/JNEUROSCI.4805-06.2007 17392476 PMC6672107

[97] Keyes BE, Fuchs E. Stem cells: Aging and transcriptional fingerprints. J Cell Biol. 2018; 217:79–92. 10.1083/jcb.201708099 29070608 PMC5748991

[98] Lee B, Song YS, Rhodes C, Goh TS, Roh JS, Jeong H, Park J, Lee HN, Lee SG, Kim S, Kim M, Lee SI, Sohn DH, Robinson WH. Protein phosphatase magnesium-dependent 1A induces inflammation in rheumatoid arthritis. Biochem Biophys Res Commun. 2020; 522:731–5. 10.1016/j.bbrc.2019.11.112 31791585 PMC7218691

[99] Zhan L, Chen S, Li K, Liang D, Zhu X, Liu L, Lu Z, Sun W, Xu E. Autophagosome maturation mediated by Rab7 contributes to neuroprotection of hypoxic preconditioning against global cerebral ischemia in rats. Cell Death Dis. 2017; 8:e2949. 10.1038/cddis.2017.330 28726776 PMC5550874

[100] Bolz H, Ramírez A, von Brederlow B, Kubisch C. Characterization of ADAMTS14, a novel member of the ADAMTS metalloproteinase family. Biochim Biophys Acta. 2001; 1522:221–5. 10.1016/s0167-4781(01)00329-3 11779638

[101] Zhang Z, Chen B, Zhu Y, Zhang T, Zhang X, Yuan Y, Xu Y. Corrigendum: The Jumonji Domain-Containing Histone Demethylase Homolog 1D/lysine Demethylase 7A (JHDM1D/KDM7A) Is an Epigenetic Activator of RHOJ Transcription in Breast Cancer Cells. Front Cell Dev Biol. 2021; 9:729416. 10.3389/fcell.2021.729416 34395453 PMC8356478

[102] Yang X, Wang G, Wang Y, Zhou J, Yuan H, Li X, Liu Y, Wang B. Histone demethylase KDM7A reciprocally regulates adipogenic and osteogenic differentiation via regulation of C/EBPα and canonical Wnt signalling. J Cell Mol Med. 2019; 23:2149–62. 10.1111/jcmm.14126 30614617 PMC6378189

[103] Tian M, Huang Y, Song Y, Li W, Zhao P, Liu W, Wu K, Wu J. MYSM1 Suppresses Cellular Senescence and the Aging Process to Prolong Lifespan. Adv Sci (Weinh). 2020; 7:2001950. 10.1002/advs.202001950 33240758 PMC7675055

[104] Sheffield DA, Jepsen MR, Feeney SJ, Bertucci MC, Sriratana A, Naughtin MJ, Dyson JM, Coppel RL, Mitchell CA. The myotubularin MTMR4 regulates phagosomal phosphatidylinositol 3-phosphate turnover and phagocytosis. J Biol Chem. 2019; 294:16684–97. 10.1074/jbc.RA119.009133 31543504 PMC6851315

