# Supplementary material for: Hallmarks of aging-based dual-purpose disease and age-associated targets predicted using PandaOmics AI-powered discovery engine
Source: Aging (Albany NY). 2022 Mar 29;14(6):2475–506. doi: 10.18632/aging.203960 (PMC9004567; doi:10.18632/aging.203960)
Supplement: Supplementary Figures [file aging-14-203960-s001.pdf]

SUPPLEMENTARY FIGURES

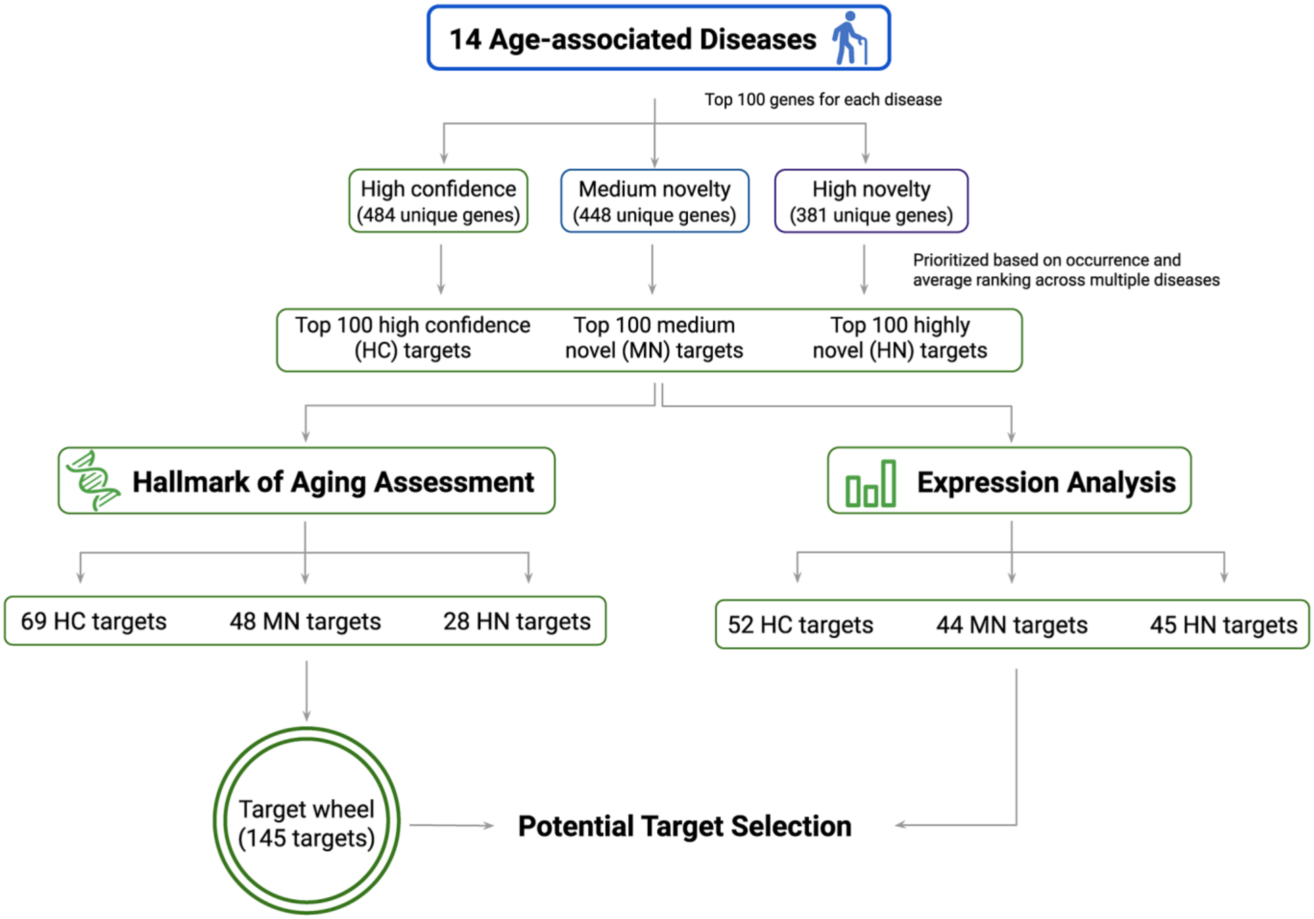

Supplementary Figure 1. Flowchart of the selection of dual-purpose targets from the 14 AADs. Upon target identifications from the 14 AADs, 484 high confidence (HC) targets, 448 medium novel (MN) targets and 381 highly novel (HN) targets were identified by PandaOmics. Targets ranked as top 100 for each novelty (total 300 targets) were subjected to the hallmarks of aging assessment by searching the literature for their evidence in modulating longevity or longevity pathways, and consistency in dysregulated expression across disease classes. A total of 145 targets including 69 HC targets, 48 MN targets and 28 HN targets were associated with aging hallmarks whereas 52 HC targets, 44 MN targets and 45 HN targets were consistently dysregulated in two or more disease classes in a unidirectional manner. Potential dual-purpose candidates were selected with reference to both the hallmarks of aging assessment and expression analysis.

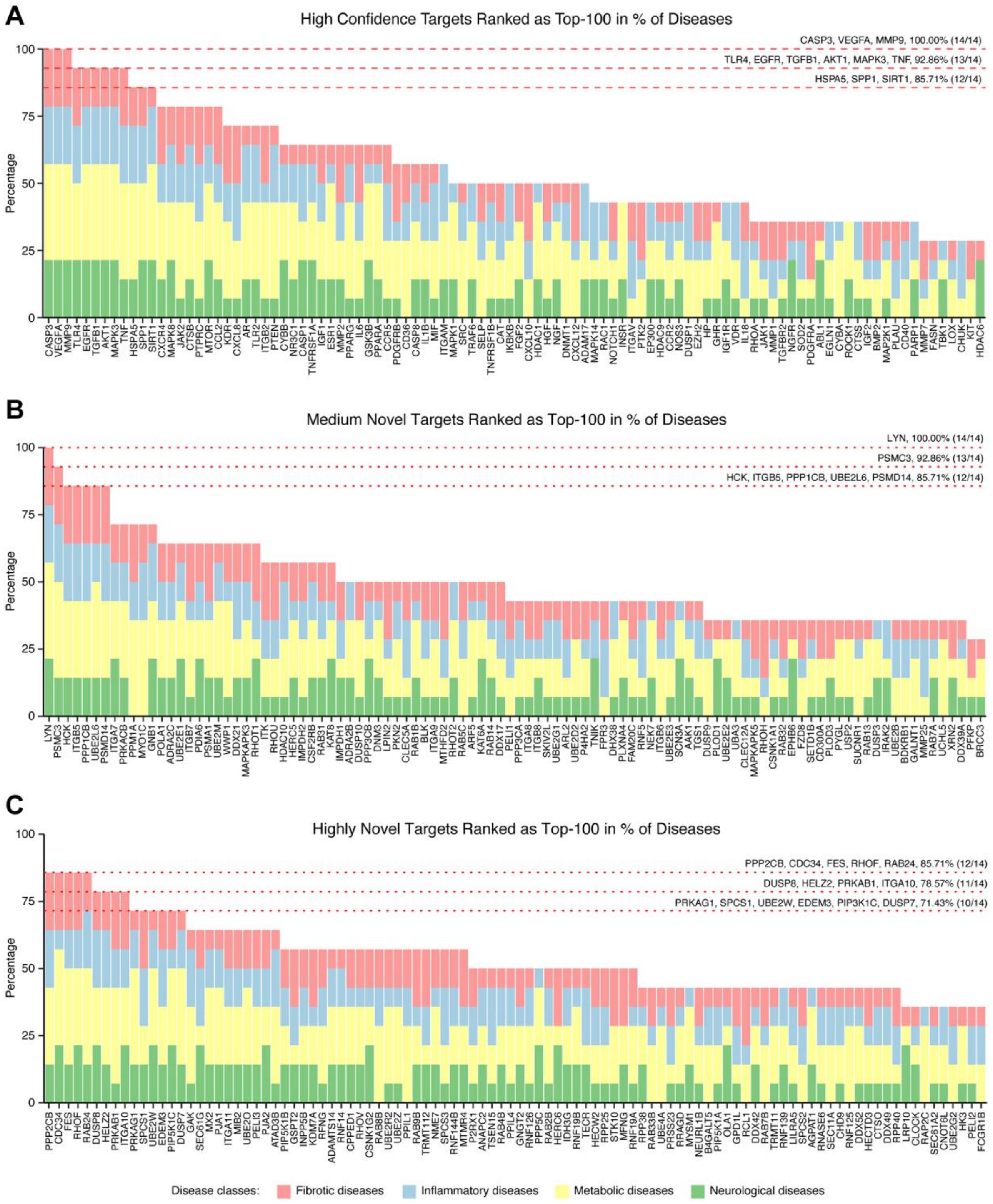

Supplementary Figure 2. Occurrence of the top-100 targets in the 14 AADs. The y-axis indicates the percentage of diseases in which the target was highly ranked (≤100) under (**A**) high confidence, (**B**) medium novel, and (**C**) highly novel filter settings. The targets with the highest percentages are exemplified above the horizontal dashed lines with their occurrence percentages shown in brackets. AADs are colored according to their disease classes.

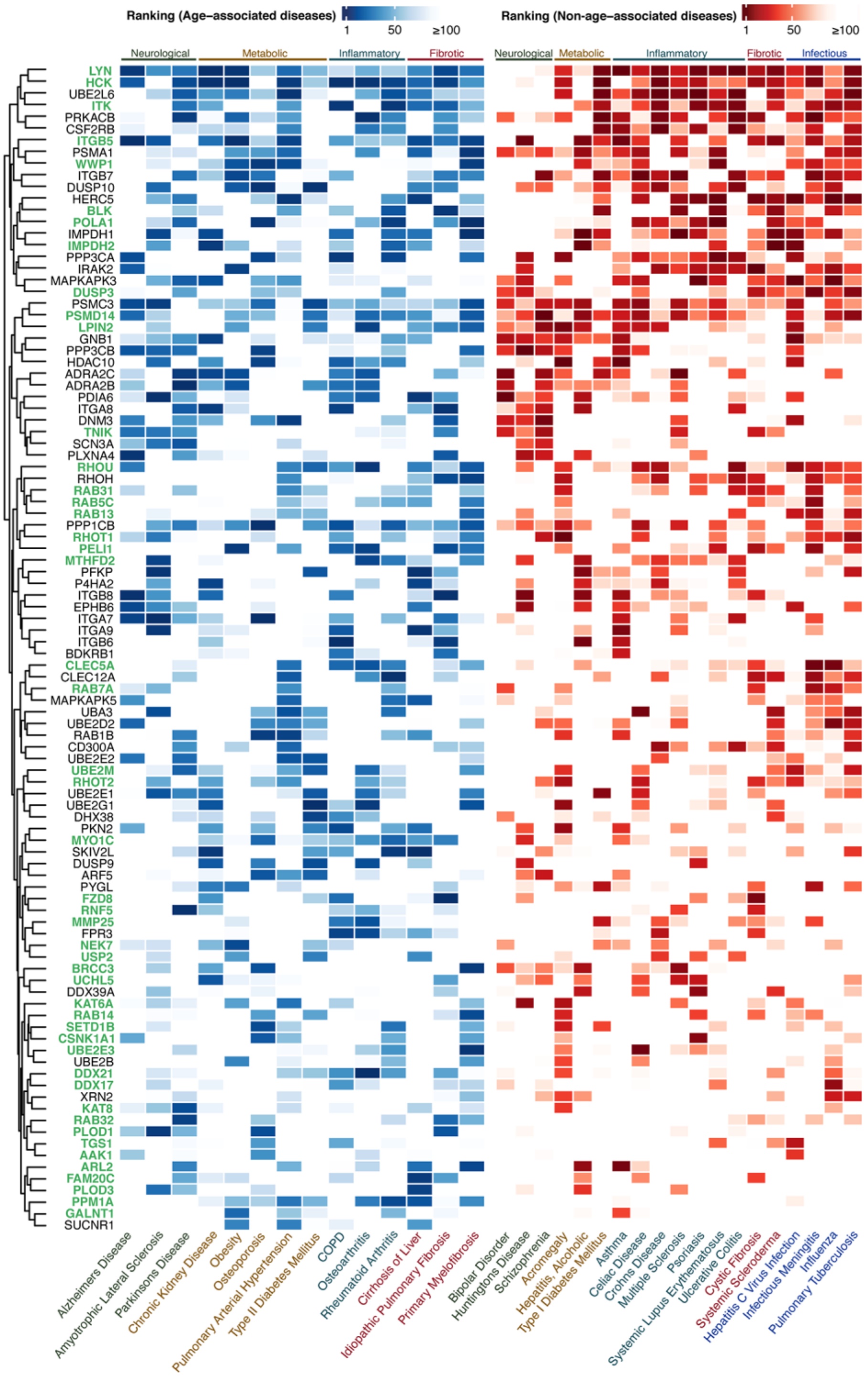

Supplementary Figure 3. Ranking of the top-100 gene set for AADs under medium novelty settings. The ranking of the targets in AADs or NAADs were colored in blue-white and red-white thermal scales respectively. High color intensity stands for high rankings. The lowest ranking was capped at 100. Targets associated with the hallmark(s) of aging are labeled in green. AADs and NAADs are colored according to their disease classes.

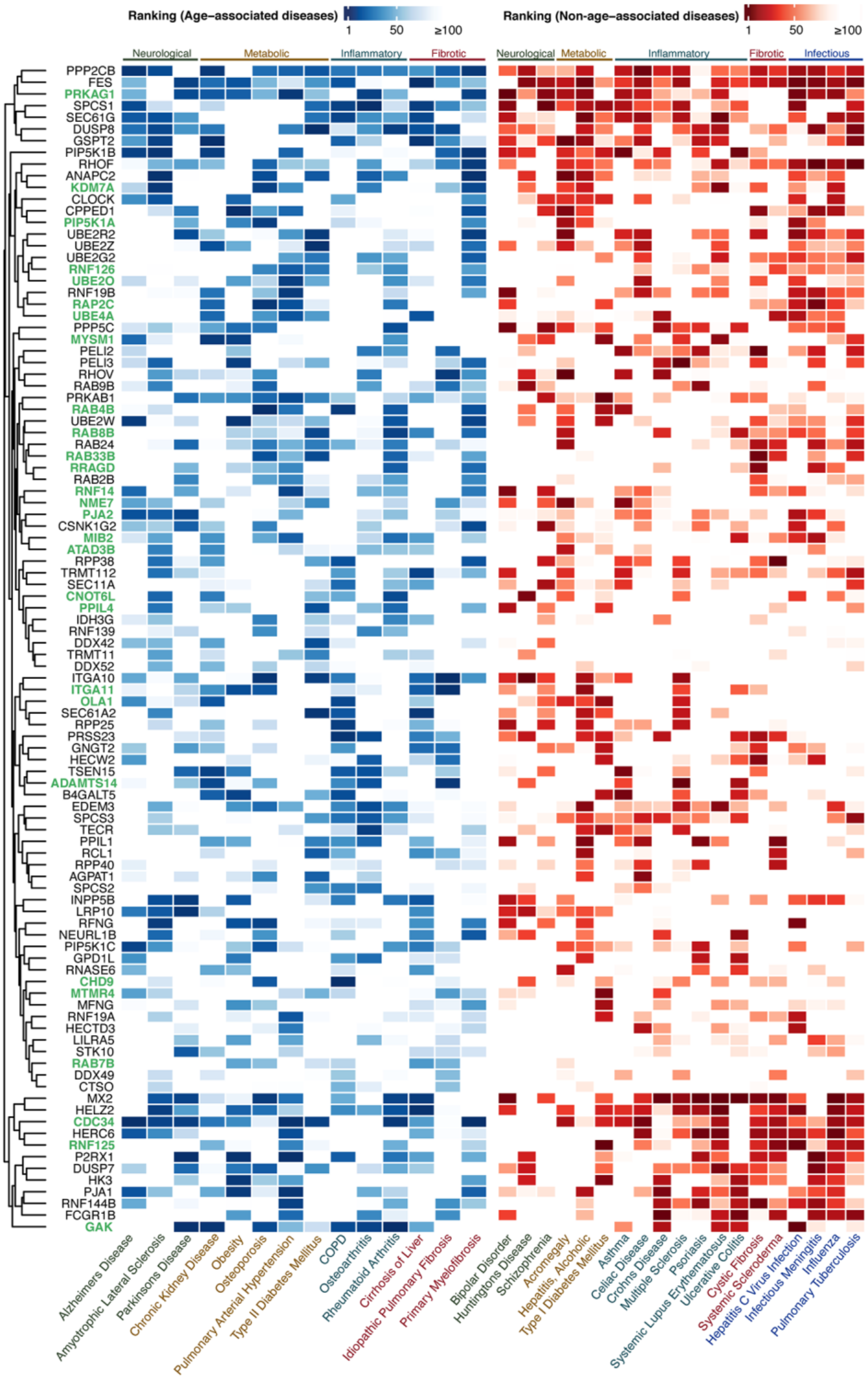

Supplementary Figure 4. Ranking of the top-100 gene set for AADs under high novelty settings. The ranking of the targets in AADs or NAADs were colored blue-white and red-white thermal scales respectively. High color intensity stands for high rankings. The lowest ranking was capped at 100. Targets associated with the hallmark(s) of aging are labeled in green. AADs and NAADs are colored according to their disease classes.

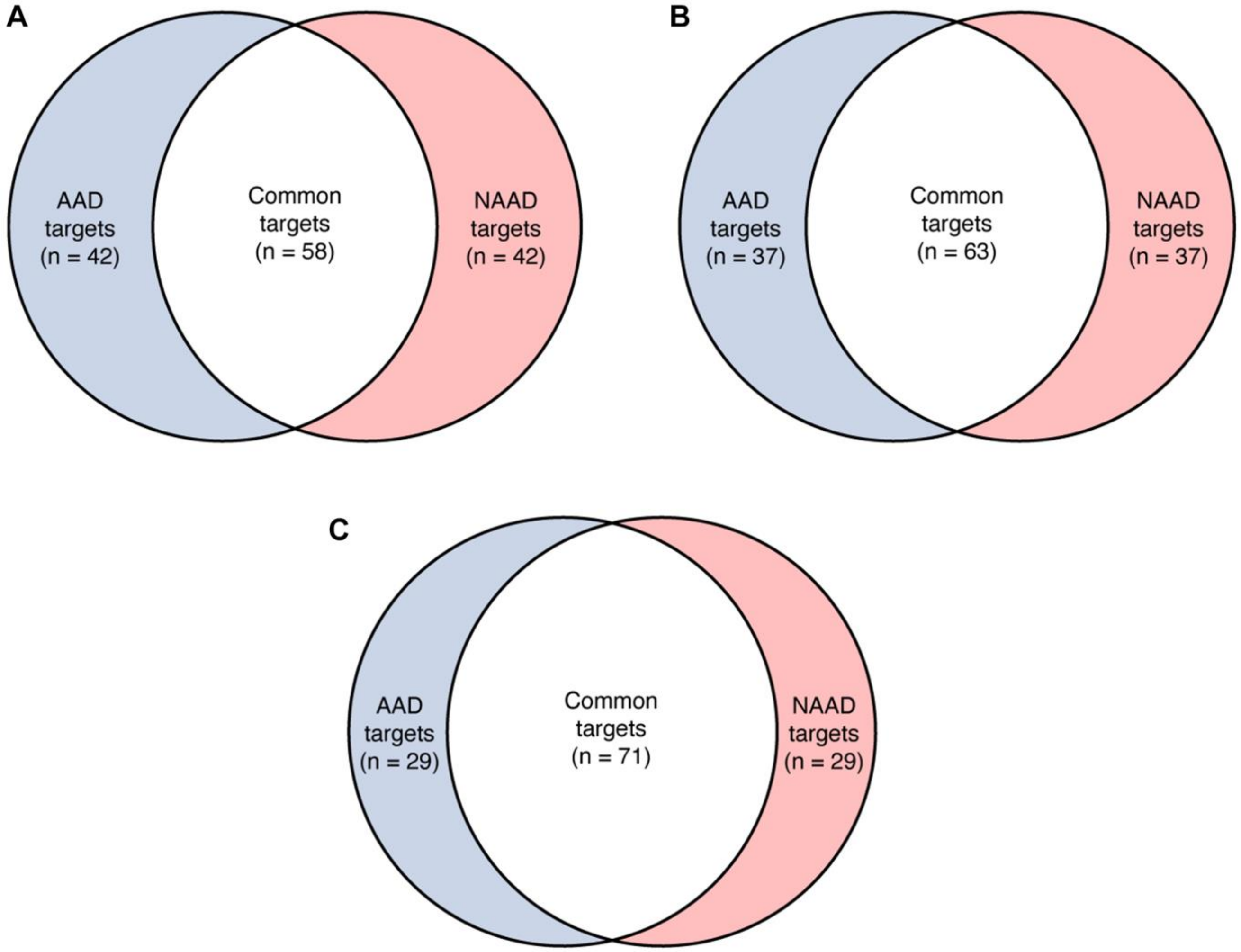

Supplementary Figure 5. Overlapping between the two sets of top-100 genes from AADs and NAADs. Top-ranked targets shared by both AAD and NAAD categories were regarded as common targets, while targets unique to AADs were defined as AAD targets under (**A**) high confidence, (**B**) medium novelty, and (**C**) high novelty filter settings.
