# Supplementary material for: Hallmarks of aging-based dual-purpose disease and age-associated targets predicted using PandaOmics AI-powered discovery engine
Source: Aging (Albany NY). 2022 Mar 29;14(6):2475–506. doi: 10.18632/aging.203960 (PMC9004567; doi:10.18632/aging.203960)
Supplement: Supplementary Tables 3-5 and 8 [file aging-14-203960-s003.pdf]

SUPPLEMENTARY TABLES

Please browse Full Text version to see the data of Supplementary Tables 1, 2, 6 and 7.

Supplementary Table 1. Top-100 genes for each filter setting.

Supplementary Table 2. Genes associated with hallmarks of aging.

Supplementary Table 3. Overlapping of high confidence targets with the pool of curated aging-associated genes from clinical trials.

Gene[1]	Aging clinical trial[2]	Druggability[3]	Target family[4]	Top-100 target[5]
ABL1	DASATINIB (NCT04994561, NCT04946383)	2,0,2,0	Tyrosine kinase	✓
AR	TESTOSTERONE (NCT00182871, NCT00309855, NCT00680797, NCT02203656, NCT02679274, NCT02990533)	2,0,2,0	Nuclear receptor	✓
ESR1	CLIMARA (NCT00220454, NCT02042196)	2,2,2,0	Nuclear receptor	✓
GHR	GROWTH HORMONE RELEASING HORMONE (GHRH) (NCT01410799)	2,2,2,0	Immunoglobulin	✓
IGF1	ORALLY ACTIVE GROWTH HORMONE SECRETAGOGUE (MK-677) (NCT00474279)	2,2,2,0	Growth factor	✓
IGF1R	INSULIN-LIKE GROWTH FACTOR 1 (NCT03932162)	2,2,2,0	Receptor kinase	✓
KIT	DASATINIB (NCT04994561, NCT04946383)	2,2,2,0	Receptor kinase	✓
MAPK14	DASATINIB (NCT04994561, NCT04946383)	2,0,1,0	CMGC kinase	✓
MTOR	RAPAMYCIN (NCT02874924, NCT04488601, NCT04742777)	2,0,2,0	Protein kinase	✓
NR3C1	METHYLPREDNISOLONE (NCT03529929)	2,0,2,0	Nuclear receptor	✓
PDGFRB	DASATINIB (NCT04994561, NCT04946383)	2,2,2,0	Receptor kinase	✓
SIRT1	RESVERATROL (NCT02523274)	2,0,2,0	Acyltransferase	✓
SRC	DASATINIB (NCT04994561, NCT04946383)	2,2,2,0	Tyrosine kinase	✓
VDR	NT-020 (NCT01963767)	2,0,2,0	Nuclear receptor	✓
ACE	PERINDOPRIL (NCT03295734)	2,2,2,0	Glycosylase	
AGTR1	CANDESARTAN (NCT00605072)	2,2,2,0	GPCR	
AMY2A	ACARBOSE (NCT02865499, NCT02953093)	2,0,2,0	Glycosylase	
BCR	DASATINIB (NCT04994561, NCT04946383)	2,2,1,0	Protein kinase	
BST1	NICOTINAMIDE MONONUCLEOTIDE (NCT04823260)	2,2,2,0	Glycosylase	
BTK	DASATINIB (NCT04994561, NCT04946383)	2,2,2,0	Tyrosine kinase	
CHRNA3	NICOTINE PATCH, ORAL MECAMYLAMINE, PLACEBO (NCT03408574)	2,0,1,0	Ion channel	
CHRNA4	NICOTINE PATCH, ORAL MECAMYLAMINE, PLACEBO (NCT03408574)	2,0,2,0	Ion channel	
CSK	DASATINIB (NCT04994561, NCT04946383)	2,0,2,0	Tyrosine kinase	
EPHA2	DASATINIB (NCT04994561, NCT04946383)	2,2,2,0	Receptor kinase	
EPHA5	DASATINIB (NCT04994561, NCT04946383)	2,2,1,0	Receptor kinase	
EPHB4	DASATINIB (NCT04994561, NCT04946383)	2,2,2,0	Receptor kinase	
ETFDH	METFORMIN (NCT01765946, NCT02308228, NCT02432287, NCT03072485, NCT03309007, NCT03451006, NCT03713801, NCT04264897)	2,0,1,0	Oxidoreductase	
FGR	DASATINIB (NCT04994561, NCT04946383)	2,1,2,2	Tyrosine kinase	
FKBP1A	RAPAMYCIN (NCT02874924, NCT04488601, NCT04742777)	2,0,2,0	Isomerase	
FRK	DASATINIB (NCT04994561, NCT04946383)	2,0,1,2	Tyrosine kinase	

FYN	DASATINIB (NCT04994561, NCT04946383)	2,2,2,0	Tyrosine kinase
GAA	ACARBOSE (NCT02865499, NCT02953093)	2,0,2,0	Glycosylase
GABRA1	ZOLPIDEM (NCT00383357, NCT03657212)	2,0,2,0	Ion channel
GABRA2	ZOLPIDEM (NCT00383357, NCT03657212)	2,0,2,0	Ion channel
GABRA3	ZOLPIDEM (NCT00383357, NCT03657212)	2,0,2,1	Ion channel
GABRB1	ZOLPIDEM (NCT00383357, NCT03657212)	2,0,1,1	Ion channel
GABRG2	ZOLPIDEM (NCT00383357, NCT03657212)	2,0,2,0	Ion channel
GHSR	ORALLY ACTIVE GROWTH HORMONE SECRETAGOGUE (MK-677) (NCT00474279)	2,0,2,0	GPCR
GPD1	METFORMIN (NCT01765946, NCT02308228, NCT02432287, NCT03072485, NCT03309007, NCT03451006, NCT03713801, NCT04264897)	1,0,1,0	Oxidoreductase
GPD2	METFORMIN (NCT01765946, NCT02308228, NCT02432287, NCT03072485, NCT03309007, NCT03451006, NCT03713801, NCT04264897)	2,0,1,0	Oxidoreductase
LCK	DASATINIB (NCT04994561, NCT04946383)	2,2,2,0	Tyrosine kinase
MGAM	ACARBOSE (NCT02865499, NCT02953093)	2,0,2,0	Glycosylase
NR3C2	TESTOSTERONE (NCT00182871)	2,0,2,0	Nuclear receptor
NR4A3	DASATINIB (NCT04994561, NCT04946383)	1,0,1,0	Nuclear receptor
OXTR	OXYTOCIN NASAL SPRAY (NCT03119610)	2,2,2,0	GPCR
PPAT	DASATINIB (NCT04994561, NCT04946383)	2,0,2,0	Glycosyltransferase
PTGS1	DICLOFENAC (NCT03072485)	2,0,2,0	Oxidoreductase
PTGS2	DICLOFENAC (NCT03072485)	2,2,2,0	Oxidoreductase
SI	ACARBOSE (NCT02865499, NCT02953093)	2,0,1,0	Glycosylase
SRD5A1	DUTASTERIDE (NCT00309855)	2,0,2,0	Oxidoreductase
SRD5A2	DUTASTERIDE (NCT00309855)	2,0,1,0	Oxidoreductase
SRMS	DASATINIB (NCT04994561, NCT04946383)	2,0,1,0	Tyrosine kinase
TERT	AAV-HTERT (NCT04133649)	2,2,2,0	Transferase
YES1	DASATINIB (NCT04994561, NCT04946383)	2,2,2,0	Tyrosine kinase
ABL2	DASATINIB (NCT04994561, NCT04946383)	1,0,1,1	Tyrosine kinase
CHRNB2	NICOTINE PATCH, ORAL MECAMYLAMINE, PLACEBO (NCT03408574)	2,0,2,1	Ion channel
CHRNB4	NICOTINE PATCH, ORAL MECAMYLAMINE, PLACEBO (NCT03408574)	2,0,2,1	Ion channel
SRD5A3	DUTASTERIDE (NCT00309855)	2,2,1,1	Oxidoreductase
BLK*	DASATINIB (NCT04994561, NCT04946383)	2,0,2,1	Tyrosine kinase
HCK*	DASATINIB (NCT04994561, NCT04946383)	2,2,2,1	Tyrosine kinase
LYN*	DASATINIB (NCT04994561, NCT04946383)	2,2,2,1	Tyrosine kinase
PRKAB1^	METFORMIN (NCT01765946, NCT02308228, NCT02432287, NCT03072485, NCT03309007, NCT03451006, NCT03713801, NCT04264897)	2,0,1,2	Protein kinase

[1]Curated pool of aging-associated genes (genes identified as medium novel targets were marked with asterisks, and highly novel targets with arrow heads) Sources of curation was http://ClinicalTrials.gov aging drug targets. [2]Example of drug investigated in aging clinical trials with clinical trial ID shown in parenthesis. Target-drug association were manually curated. [3]Druggability scores defined in PandaOmics (small molecules, antibodies, safety, novelty). [4]Druggable gene classes defined in PandaOmics. [5]Genes identified as top-100 high-confidence targets are marked with ticks.

Supplementary Table 4. Overlapping of high confidence targets with the pool of curated aging-associated genes from publication.

Genes[1]	Gene Name[2]	Druggability[3]	Target family[4]	Top-100 target[5]
AKT1	AKT serine/threonine kinase 1	2,0,2,0	AGC kinase	✓
CASP3	caspase 3	2,0,2,0	Peptidase	✓
CAT	catalase	2,0,2,0	Oxidoreductase	✓
CHUK	component of inhibitor of nuclear factor kappa B kinase complex	2,0,2,0	Protein kinase	✓
DNMT1	DNA methyltransferase 1	2,0,1,0	Methyltransferase	✓
EGFR	epidermal growth factor receptor	2,2,2,0	Receptor kinase	✓
HDAC9	histone deacetylase 9	2,0,1,0	Hydrolase	✓
IGF1	insulin like growth factor 1	2,2,2,0	Growth factor	✓
IGF1R	insulin like growth factor 1 receptor	2,2,2,0	Receptor kinase	✓
IL1B	interleukin 1 beta	2,2,2,0	Interleukin	✓
IL6	interleukin 6	2,2,2,0	Interleukin	✓
JAK2	Janus kinase 2	2,0,2,0	Tyrosine kinase	✓
MAPK8	mitogen-activated protein kinase 8	2,0,2,0	CMGC kinase	✓
MMP1	matrix metallopeptidase 1	2,2,2,0	Peptidase	✓
MMP2	matrix metallopeptidase 2	2,2,2,0	Peptidase	✓
MMP9	matrix metallopeptidase 9	2,2,2,0	Peptidase	✓
MTOR	mechanistic target of rapamycin kinase	2,0,2,0	Protein kinase	✓
PPARA	peroxisome proliferator activated receptor alpha	2,0,2,0	Nuclear receptor	✓
PTEN	phosphatase and tensin homolog	1,1,1,0	Esterase	✓
SIRT1	sirtuin 1	2,0,2,0	Acyltransferase	✓
SOD2	superoxide dismutase 2	1,0,1,0	Oxidoreductase	✓
TGFB1	transforming growth factor beta 1	2,2,2,0	Growth factor	✓
TGFBR2	transforming growth factor beta receptor 2	2,2,2,0	Receptor kinase	✓
TNF	tumor necrosis factor	2,2,2,0	Tumor necrosis factor	✓
ABO	ABO, alpha 1-3-N-acetylgalactosaminyltransferase and alpha 1-3-galactosyltransferase	1,0,1,0	Glycosyltransferase	
AKT2	AKT serine/threonine kinase 2	2,0,2,0	AGC kinase	
BMP1	bone morphogenetic protein 1	1,0,1,0	Peptidase	
GZMB	granzyme B	2,0,2,0	Peptidase	
HAS1	hyaluronan synthase 1	1,2,1,0	Glycosyltransferase	
HAS2	hyaluronan synthase 2	1,0,1,0	Glycosyltransferase	
HDAC11	histone deacetylase 11	2,2,2,0	Hydrolase	
HDAC4	histone deacetylase 4	2,0,2,0	Hydrolase	
IL15	interleukin 15	2,2,2,0	Interleukin	
MME	membrane metalloendopeptidase	2,2,2,0	Peptidase	
MMP13	matrix metallopeptidase 13	2,2,2,0	Peptidase	
MT-ND2	mitochondrially encoded NADH:ubiquinone oxidoreductase core subunit 2	2,0,1,0	Translocase	
NOX4	NADPH oxidase 4	2,0,2,0	Oxidoreductase	
PPIA	peptidylprolyl isomerase A	2,0,2,0	Isomerase	
PRDX2	peroxiredoxin 2	1,0,1,0	Oxidoreductase	
PRKCD	protein kinase C delta	2,0,2,0	AGC kinase	

PTGS2	prostaglandin-endoperoxide synthase 2	2,2,2,0	Oxidoreductase
SIRT6	sirtuin 6	2,0,1,0	Acyltransferase
SOD1	superoxide dismutase 1	2,2,2,0	Oxidoreductase
TYR	tyrosinase	2,0,2,0	Oxidoreductase
WNK2	WNK lysine deficient protein kinase 2	1,0,1,0	Protein kinase
XDH	xanthine dehydrogenase	2,0,2,0	Oxidoreductase
AOPEP	aminopeptidase O (putative)	2,0,1,2	Peptidase
CLOCK^	clock circadian regulator	1,0,1,2	Acyltransferase

[1]Curated pool of aging-associated genes (genes identified as medium novel targets are marked with asterisks, and highly novel targets with arrow heads). [2]These genes were associated with aging or skin aging with reference to a publicity database. [3]Druggability scores defined in PandaOmics (small molecules, antibodies, safety, novelty). [4]Druggable gene classes defined in PandaOmics. [5]Genes identified as top-100 high confidence targets were marked with ticks.

Supplementary Table 5. Overlapping of high confidence targets with the pool of curated aging-associated genes from geroprotectors.

Genes[1]	Geroprotectors[2]	Druggability[3]	Target family[4]	Top-100 target[5]
CASP1	Aspirin	2,0,2,0	Peptidase	✓
CASP3	Aspirin	2,0,2,0	Peptidase	✓
CHUK	N-acetyl-L-cysteine	2,0,2,0	Protein kinase	✓
ESR1	17-A-Estradiol; Melatonin	2,2,2,0	Nuclear receptor	✓
HSPA5	Aspirin	2,2,2,0	Hydrolase	✓
IKBKB	Aspirin; N-acetyl-L-cysteine	2,0,2,0	Protein kinase	✓
MTOR	Rapamycin	2,0,2,0	Protein kinase	✓
ACE	Enalapril	2,2,2,0	Glycosylase	
ACY1	N-acetyl-L-cysteine	2,0,2,0	Hydrolase	
ADRB1	Metoprolol; Nebivolol	2,2,2,0	GPCR	
ADRB2	Metoprolol; Nebivolol	2,0,2,0	GPCR	
ADRB3	Nebivolol	2,2,2,0	GPCR	
AKR1C1	Aspirin	1,0,1,0	Oxidoreductase	
ALOX5	Nordihydroguaiaretic Acid	2,0,2,0	Oxidoreductase	
AMY2A	Acarbose	2,0,2,0	Glycosylase	
ASMT	Melatonin	1,0,1,0	Methyltransferase	
CHRNA4	17-A-Estradiol	2,0,2,0	Ion channel	
CKB	Creatine	2,0,1,0	Unclassified kinase	
CKM	Creatine	2,0,1,0	Non-protein kinase	
EDNRA	Aspirin	2,2,2,0	GPCR	
EPX	Melatonin	1,0,1,0	Oxidoreductase	
ESR2	17-A-Estradiol	2,0,2,0	Nuclear receptor	
ETFDH	Metformin	2,0,1,0	Oxidoreductase	
GAA	Acarbose	2,0,2,0	Glycosylase	
GAMT	Creatine	2,0,1,0	Methyltransferase	
GPD1	Metformin	1,0,1,0	Oxidoreductase	
GPER1	17-A-Estradiol	1,0,1,0	GPCR	
GRIN1	N-acetyl-L-cysteine	2,0,1,0	Ion channel	
GRIN2A	N-acetyl-L-cysteine	2,0,1,0	Ion channel	
GRIN2B	N-acetyl-L-cysteine	2,0,2,0	Ion channel	

GRIN2D	N-acetyl-L-cysteine	2,0,1,0	Ion channel
GRIN3A	N-acetyl-L-cysteine	2,0,1,0	Ion channel
GSS	N-acetyl-L-cysteine	2,0,1,0	Ligase
IFNG	D-Glucosamine	2,2,2,0	Interferon
MAOA	Deprenyl or Selegiline	2,0,2,0	Oxidoreductase
MAOB	Deprenyl or Selegiline	2,0,2,0	Oxidoreductase
MGAM	Acarbose	2,0,2,0	Glycosylase
MPO	Melatonin	2,0,2,0	Oxidoreductase
MTNR1A	Melatonin	2,0,2,0	GPCR
MTNR1B	Melatonin	2,0,2,0	GPCR
NEU1	Aspirin	1,0,1,0	Glycosylase
NPY2R	Cysteamine	2,0,2,0	GPCR
NQO2	Melatonin	2,0,2,0	Oxidoreductase
NR1I2	17-A-Estradiol	2,0,2,0	Nuclear receptor
PTGS1	Aspirin	2,0,2,0	Oxidoreductase
PTGS2	Aspirin	2,2,2,0	Oxidoreductase
RPS6KA3	Aspirin	2,0,2,0	AGC kinase
SI	Acarbose	2,0,1,0	Glycosylase
CKMT1A	Creatine	2,0,1,1	Unclassified kinase
CKMT2	Creatine	2,0,1,1	Unclassified kinase
RORB	Melatonin	2,0,2,1	Nuclear receptor
PRKAB1^	Metformin	2,0,1,2	Protein kinase

[1]Curated pool of geroprotector-associated genes (genes identified as medium novel targets are marked with asterisks, and highly novel targets with arrow heads). [2]These geroprotectors were (1) approved drugs for human use and (2) investigated for antiaging effects using human or animal models (with reference to http://geroprotectors.org). Target-drug association were manually curated. [3]Druggability scores defined in PandaOmics (small molecules, antibodies, safety, novelty). [4]Druggable gene classes defined in PandaOmics. [5]Genes identified as top-100 high confidence targets were marked with ticks.

Supplementary Table 6. Pathway enrichment analysis based on 145 targets associated with the hallmarks of aging.

Supplementary Table 7. List of AAD and NAAD datasets analyzed.

Supplementary Table 8. The identification of target-target interactions.

Targets interactions[1]	Reference (PMID)
ROCK1-c-Myc	30613282
FOXO-SIRT3	27686535
mTOR-STAT3	26697523
mTOR-TFEB	30120233
mTOR-PPARG	27901044
MAPK14-c-Myc	10623602
MAPK8-c-Myc	10623602
MAPK-CREB	30214393
MAPK-NRF2	31221142
KDM7A-Catenin beta-1	30614617
KDM7A-Catenin beta-1	32214833

[1]Targets interactions were identified outside the context of pathways with KEGG pathway database.
